# Evaluation of Chalcone (*E*)‑3-(4-Fluorophenyl)-1-(2-hydroxy-3,4,6-trimethoxyphenyl)-prop-2-en-1-one against *Trypanosoma cruzi* (Y Strain): In Vitro Assay, In Silico Drug-Target Analysis, and MPO-Based Drug Likeness

**DOI:** 10.1021/acsomega.5c08163

**Published:** 2026-07-23

**Authors:** José Ivo Lima Pinto Filho, Francisco Nithael Melo Lucio, Lyanna Rodrigues Ribeiro, Matheus Nunes da Rocha, Hélcio Silva dos Santos, Francisco Wagner de Queiroz Almeida-Neto, Emanuel Paula Magalhaes, Alice Maria Costa Martins, Ramon Róseo Paula Pessoa Bezerra de Menezes, Márcia Machado Marinho, Pedro De Lima-Neto, Emmanuel Silva Marinho

**Affiliations:** † Department of Analytical Chemistry and Physical Chemistry, Federal University of Ceará, Fortaleza, Ceará 60355-636, Brazil; ‡ Doctoral Program in Biotechnology, Northeast Biotechnology Network, State University of Ceará, Fortaleza, Ceará 60714-903, Brazil; § Department of Clinical and Toxicological Analysis, Federal University of Ceará, Fortaleza, Ceará 60355-636, Brazil; ∥ Postgraduate Program in Natural Sciences, Sciences and Technology Center, State University of Ceará, Fortaleza, Ceará 60714-903, Brazil; ⊥ Center for Exact Sciences and Technology, State University of Vale do Acaraú, Sobral, Ceará 62040-370, Brazil; # Center for Education, Science and Technology of the Inhamuns Region, State University of Ceará, Tauá, Ceará 60714-903, Brazil; ¶ Postgraduate Program in Veterinary Sciences, State University of Ceará, Fortaleza, Ceará 60714-903, Brazil; ∇ Professional Postgraduate Program in Biotechnology in Human and Animal Health, State University of Ceará, Fortaleza, Ceará 60714-903, Brazil

## Abstract

Chagas disease (CD), once found mainly in underdeveloped countries, is becoming a public health problem in the developed world. Although the drug benznidazole (BZN) is effective in the acute phase of the disease, it causes toxicity due to the formation of reactive substances resulting from presystemic metabolism, which have the ability to bind to DNA structures. This study conducts experimental tests with the epimastigote and trypomastigote species of the parasite, followed by drug–target interaction analyses through molecular docking against the enzymes trypanothione reductase, cruzain, and TcGAPDH, as well as pharmacokinetic prediction based on MPO analyses. In vitro tests revealed CPN4F’s significant efficacy in reducing host cell viability and inhibiting parasite growth. Molecular docking indicated CPN4F’s favorable energy ordering and superiority to BZN against the cruzain target (Δ*G* < −6.0 kcal mol^–1^), while molecular dynamics simulations showed that the complex remains stable in the 500 ns range. Pharmacokinetic estimates suggested high cell permeability (*P*_app_ > 10 × 10^–6^ cm/s) but potential metabolic stability concerns (CL_int,u_ > 8 mL/min/kg), showing good oral bioavailability, although with moderate metabolism. The CPN4F molecule demonstrates potent in vitro efficacy against Chagas disease, outperforming BZN in molecular docking studies targeting cruzain. Despite concerns about metabolic stability due to its high cell permeability and lipophilic nature, CPN4F exhibits low acute oral toxicity, highlighting its potential as a safe and effective treatment option.

## Graphical abstract

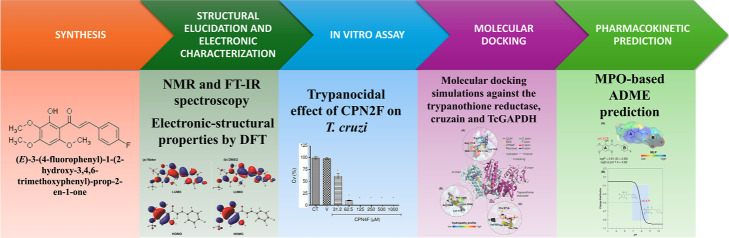

## 1 Introduction

Chagas disease (CD), also known as trypanosomiasis, is a tropical disease that affects at least 21 Latin American countries. The disease is caused by a parasite called *Trypanosoma cruzi*, which is transmitted by infected insects, such as triatomines. CD mainly affects regions with poor living conditions and low quality of life.^1–4^

The disease causes heart failure in humans and is a neglected endemic that infects around 6 to 7 million people annually worldwide. According to the World Health Organization (WHO), it kills around 10,000 people each year, which is approximately 20–30% of those affected. The difficulty in diagnosing and treating the disease is the reason for the high mortality rate.^3,5,6^ The disease is endemic in South America, but because of urbanization and population emigration, its epidemiological prevalence has changed. This has affected many countries in North America, Europe, and Africa.^7,8^

In recent years, antibiotics and vaccines have been developed to prevent the spread and exacerbation of many types of infectious diseases around the world.^9,10^ The most emerging form of treatment for the disease is the use of oral drugs with trypanocidal action.^11^ Benznidazole (BZN), a trypanocidal drug approved to treat Chagas disease since the 1960s, exerts its action by inhibiting the development of the parasite through covalent binding to essential target proteins in its evolutionary cycle.^12,13^

Despite its efficacy in the acute phase of the disease, the compound is toxic due to the formation of reactive substances from presystemic metabolism that have the ability to covalently bind to DNA structures.^12–14^ BZN is an organic compound with nitrogen atoms in its heterocyclic structure. This pharmacophore is considered essential for the development of new drugs with similar or even more effective trypanocidal activity, but with a lower risk of toxicity to the host organism.^15^

Previously endemic only in underdeveloped countries, CD has become a public health problem in developed countries.^16^ However, CD remains a neglected disease whose existing therapy is limited and has significant toxicity, jeopardizing patient adherence to treatment.^17^ It is therefore necessary to develop new molecules with trypanocidal potential, with chalcones being interesting candidates in this context, given their ease of synthesis, structural versatility, and diversity of documented biological activity, including against trypanosomatids of the *Leishmania*sp. and *Trypanosoma* sp. genera.^18–20^

Checking the activity of key metabolic pathways helps to identify lead compounds that are more effective at stopping the life cycle of the parasite *T. cruzi* in Chagas disease.^21^ Trypanothione reductase is an enzyme that helps to process trypanothione, a type of molecule found in some parasites like *Trypanosoma* sp. and *Leishmania* sp. Humans do not have this enzyme, but it is similar to another enzyme called glutathione reductase. This makes it an important part of finding more specific inhibitors.^22^ GAPDH speeds up the process of converting d-glyceraldehyde-3-phosphate to 1,3-diphosphoglycerate by using NAD^+^. This happens in the presence of the cofactor, which varies in the location of its catalytic site in *Trypanosoma brucei* and *Leishmania mexicana* species in relation to human GAPDH. This helps to identify the natural substances and inhibitors of *T. cruzi* GAPDH.^23^ Cruzain, on the other hand, is a cysteine protease, a nonstructural protein that plays a key role in the degradation of proteins that are essential for the parasite’s nutrition and in weakening the host cell’s defense mechanisms.^24^

Chalcones are compounds derived from flavonoids and have an open-chain structure and an arrangement of three α,β-unsaturated carbons conjugated to a ketone carbonyl.^25^ The radical substitutions on its A and B rings, linked by this conjugated unsaturated system, are responsible for the wealth of biological activities of this pharmacophore, especially in the development of promising new antimicrobials with antibacterial,^26,27^ antifungal,^28,29^ antiviral,^30,31^ and antiparasitic^32,33^ activities. Scientific evidence shows that the addition of fluoro-substituted groups can lead to a significant improvement in the cell permeability of small molecules,^34^ favoring the development of new drugs with more viable pharmacokinetic properties.

Halogens (F, Cl, and Br) can have positive effects on the pharmacokinetics of small molecules. They can improve metabolic stability and increase the permeability in cell membranes. However, the impact of this group on a chalcone depends on several factors such as the position of the atom in the molecule and its intramolecular interactions.^35,36^

The aim of this study is to evaluate the trypanocidal effect of a semisynthetic fluorochalcone on a partially resistant strain of *T. cruzi*, as well as investigate how the compound interacts with the target proteins of the parasite’s evolutionary cycle using in silico techniques to estimate pharmacokinetics and pharmacodynamics (PK/PD).

## 2 Methodology

### 2.1 Synthesis of the Chalcone (*E*)-3-(4-Fluorophenyl)-1-(2-hydroxy-3,4,6-trimethoxyphenyl)-prop-2-en-1-one

Initially, 2-hydroxy-3,4,6-trimethoxyacetophenone (2 mmol) was dissolved in 8 mL of ethanol, and a 50% sodium hydroxide (NaOH) solution was added to the mixture. After a few minutes, 4-fluorobenzaldehyde (2 mmol) was introduced, as illustrated in Figure 1. The reaction was allowed to proceed at room temperature for 24 h. At the end of the reaction, a 1:1 hydrochloric acid (HCl) solution was used to precipitate the product. The resulting product was then filtered by using a Buchner funnel.

**Figure 1:**
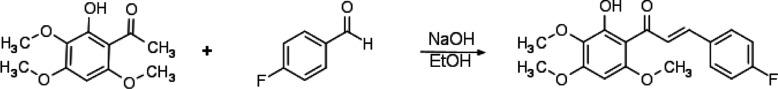
Synthesis scheme for the chalcone: (*E*)-3-(4-fluorophenyl)-1-(2-hydroxy-3,4,6-trimethoxyphenyl)-prop-2-en-1-one.

### 2.2 Nuclear Magnetic Resonance Spectroscopy

The ^1^H and ^13^C nuclear magnetic resonance (NMR) spectra for the CPN4F molecule were obtained in a Bruker DRX-300 and DPX-500 spectrometer (^1^H: 300 and 500 MHz; ^13^C: 75 and 125 MHz), using a deuterated solvent (CDCl_3_).

### 2.3 Infrared Spectroscopy

The infrared spectrum of the CPN4F molecule was obtained using Fourier transform infrared (FT-IR) spectroscopy with an IRTrazer-100 from Shimadzu (Kyoto, Japan). The spectrum was recorded over 64 scans in the wavenumber range of 4000 to 400 cm^–1^ with a resolution of 4 cm^–1^.

### 2.4 Evaluation of Cytotoxicity in LLC-MK2 Cells

To assess the cytotoxicity of the molecule, as well as its selectivity for *T. cruzi*, LLC-MK2 cells (ATCC CCl-7), a lineage of monkey renal tubule epithelial cells (*Macaca mulatta*) acquired from the Rio de Janeiro Cell Bank (BCRJ), were used. The cells were grown in DMEM (Dulbecco’s modified Eagle medium, pH 7.4) supplemented with 10% FBS (Fetal Bovine Serum) and antibiotics (penicillin—200 IU/mL and streptomycin—130 mg/mL) in sterile plastic bottles (75 cm^2^) and kept in a CO_2_ oven (37.0 ± 0.3 °C, 5% CO_2_).

After reaching confluence, the medium in the bottles was removed, the cells were washed with 5 mL of sterile PBS and displaced with 1 mL of Trypsin/EDTA solution (0.25%/0.04%), incubated for 5 to 10 min at 37 °C, and inactivated with 2 mL of DMEM 10% SBF, with aliquots being transferred to new bottles at the time of maintenance.

For freezing, after washing and dislodging the cells, they were centrifuged (4000 rpm for 5 min), the medium was removed, the pellet was resuspended in the freezing solution (95% SBF and 5% sterile DMSO), and the cells were stored in liquid nitrogen.

The MTT [3-(4,5-dimethyl-2-thiazolyl)-2,5-diphenyl-2*H*-tetrazolium bromide] reduction test, previously described,^37^ was used to assess the cytotoxicity of the substances. In this test, the yellow MTT salt is endocytosed by viable cells and is reduced by intracellular enzymes to an insoluble purple salt, the formazan salt, which is solubilized after the addition of surfactants. In this way, the concentration of formazan produced is measured spectrophotometrically at 570 nm and is directly proportional to the quantity of viable cells.

To perform the assay, follow these steps: first, the confluent cultures were washed. Then, they were trypsinized and centrifuged as described above. Next, the resulting pellet was resuspended in 1 mL of DMEM. Finally, an aliquot was resuspended in trypan blue solution (0.4% w/v in PBS) in proportions of 1:10 and 1:100 for cell density quantification in a Neubauer chamber.

For the experiments, the cell density was adjusted to 10^5^ cells/mL and transferred to sterile 96-well plates (200 μL/well). The plates were incubated in a CO_2_ oven overnight, and then the wells were treated with the molecule (1000–31.25 μM). Untreated groups and cells treated with 0.5% DMSO were used as a negative control to assess the toxicity of the diluent. In addition, wells containing only the culture medium were used as a blank.

After 24 h of incubation in a CO_2_ oven, each well was washed with 100 μL of sterile PBS, and 10 μL of MTT solution (2.5 mg/mL) was added, along with 100 μL of DMEM 10% SBF. The plates were incubated in the dark at 37 °C for 4 h, and then 90 μL of SDS solution (10% m/v in 0.05 N HCl) was added. The plates were incubated for 17 h to solubilize the formazan crystals and read using spectrophotometry in a microplate reader at 570 nm. The percentage of cell viability (Cv) was determined using eq 1

1 $$Cv=\frac{\left(\left|T\right|-\left|BC\right|\right)}{\left(\left|CT\right|-\left|BC\right|\right)}\times 100\%$$

where Abs T is the absorbance of the test group, Abs BC is the absorbance of the blank, and Abs CT is the absorbance of the control group. The Cv percentages were used to estimate, by nonlinear regression (fit curve), the concentration needed to reduce cell viability by 50% (CC_50_).

### 2.5 Evaluation of Activity on Epimastigote Forms of *T. cruzi* Strain Y

The epimastigote forms (strain Y) of *T. cruzi* were provided by the Parasite Biochemistry Laboratory at the University of São Paulo (USP) and cultivated in a Liver Infusion Tryptose (LIT) medium (with NaCl 4 g/L; Na_2_HPO_4_ 12H_2_O 11.6 g/L; KCl 0.4 g/L; glucose 2.2 g/L; tryptose 5 g/L; liver infusion 5 g/L; hemin 25 mg/L; pH 7.4) supplemented with 10% SBF and antibiotics (penicillin—200 UI/mL and streptomycin—50 mg/L). The cultures were kept at 28 ± 1 °C in a Biochemical Oxygen Demand (BOD) oven in sterile bottles.^38^

The parasites were cultivated at a density of 1 × 10^6^ cells/mL and assessed daily until they reached the exponential phase of the growth curve (sixth to eighth day of cultivation) and then subcultured by transfer to a new LIT medium. To maintain the cell stock, the cultures were centrifuged (2800 rpm for 7 min), resuspended in 95% SBF and 5% sterile DMSO, and stored in liquid nitrogen.

To assess the effect of the substances in this study on the epimastigote forms, aliquots containing the parasites in the exponential phase were diluted in complete LIT medium and transferred to a Neubauer chamber, and the cell density (cells/mL) was determined.

The parasites were then transferred to sterile 96-well plates so that the initial concentration was adjusted to 1 × 10^6^ cells/mL (200 μL/well), together with chalcone (1000–31.25 μM). In addition, the cells were treated with 0.5% DMSO to assess the effect of the vehicle on Cv, and PBS was used as a negative control.

After treatment, the plates were incubated in a BOD oven, and aliquots were taken from the experimental groups after 24, 48, and 72 h to quantify the viable parasites, with typical morphology and mobility. The percentage of viability was determined in relation to the negative control group, enabling the concentration capable of inhibiting epimastigote proliferation by 50% (IC_50_) to be determined.^39^

### 2.6 Evaluation of the Effect on Trypomastigote Forms of *T. cruzi* Strain Y

The trypomastigote forms of *T. cruzi* were obtained from the infection of host cells, as described by Lima et al.^39^ To do this, LLC-MK2 cells were grown in sterile 25 cm^2^ bottles at a concentration of 1 × 10^5^ cells/mL in DMEM 10% SBF. After 48 h of incubation in a CO_2_ oven, the medium was replaced with DMEM 2% SBF without antibiotics, and the cells were infected with trypomastigotes at a rate of 20 parasites per cell.

After 72 h, the medium in the bottles was replaced and the supernatant was centrifuged (2800 rpm for 7 min) to obtain the trypomastigotes, a procedure carried out until the sixth day postinfection. Cell density was determined by counting in a Neubauer chamber, as was done for the epimastigote forms, and the trypomastigotes were used for the tests to assess the effect of the test substances and to obtain amastigote forms.

To assess the trypanocidal effect of the substances under study, 10^6^ trypomastigotes/mL were incubated with the molecule (1000–31.25 μM) in 96-well plates (200 μL/well) with DMEM 10% SBF. Untreated parasites were used as a negative control and were 100% viable. In addition, DMSO 0.5% was used as the vehicle group.

After 24 h of incubation in a CO_2_ oven, aliquots were collected from the experimental groups, and the number of parasites was determined by counting in a Neubauer chamber. The percentage of Cv was calculated similarly to the epimastigote forms, according to the equation used for the epimastigote forms, and the lethal concentration for 50% of the parasites (LC_50_) was calculated similarly to the calculation for the epimastigote forms. In addition, the selectivity index (SI) was calculated for trypomastigote forms in relation to host cells, according to eq 2

2 $$SI=\frac{{CC}_{50}}{{LC}_{50}}$$

where CC_50_ is the concentration capable of reducing the viability of host cells by 50% and LC_50_ is the lethal concentration for 50% of the parasites.

### 2.7 Quantum Chemical Calculations

Quantum chemical calculations were performed using the density functional theory (DFT) formalism and ORCA 5.0.4 ^40^ software to explore the structural and electronic properties. A geometric optimization process was performed to obtain the lowest-energy ground-state conformation using the hybrid exchange–correlation functional B3LYP and the 6-311++G­(d,p) Pople basis set.^41–44^

The ^1^H and ^13^C NMR isotropic chemical shifts were calculated by the GIAO method, using chloroform as the implicit solvent. These shifts were obtained from the theoretical screening constants of the H and C atoms, compared to those of the TMS standard, using the formulas δH = σH­(TMS) – σH­(calc) and δC = σC­(TMS) – σC­(calc). The fundamental vibrational modes with optimized geometry in the implicit solvents water and DMSO were also calculated. The theoretical frequencies of the CPN4F molecule were fitted with scaling factors of 0.983 (for <1700 cm^–1^) and 0.958 (for >1700 cm^–1^).

From the optimized structure, the isodensities of the frontier molecular orbitals highest occupied molecular orbital (HOMO) and lowest unoccupied molecular orbital (LUMO) were plotted and the energy values of the respective molecular orbitals were used to calculate the chemical descriptors of global reactivity: HOMO–LUMO energy gap (Δ*E*_GAP_, eq 3),^45^ the ionization potential (*I*, eq 4),^46^ the electron affinity (*A*, eq 5),^47^ the electronegativity (χ, eq 6),^48,49^ the global hardness (η, eq 7),^50^ the global softness (σ, eq 8),^51^ the global electrophilicity index (ω, eq 9),^52^ and the global nucleophilicity index (ε, eq 10).^53,54^

3 $${\Delta E}_{GAP}=E_{LUMO}-E_{HOMO}$$

4 $$I=-E_{HOMO}$$

5 $$A=-E_{LUMO}$$

6 $$\chi =\frac{I+A}{2}$$

7 $$\eta =\frac{I-A}{2}$$

8 $$\sigma =\frac{1}{\eta }$$

9 $$\omega =\frac{\chi^{2}}{2\eta }$$

10 $$\varepsilon =\frac{1}{\omega }$$

To investigate the chemical reactivity of the CPN4F molecule in greater detail, the electronic Fukui function (eq 11) and the condensed Fukui functions (eqs 12–14)^55,56^ were calculated. The electronic Fukui functions for nucleophilic attack (*f* ^+^), electrophilic attack (*f* ^–^), and radical attack (*f* ^0^) were calculated using Multiwfn 3.7 software.^57^ Isosurfaces were visualized with VESTA 3.4.7 software,^58^ using an isovalue of 0.000935427 (water) and 0.000946114 (DMSO) for nucleophilic attacks, 0.00099664 (water) and 0.000998117 (DMSO) for electrophilic attacks, and 0.000773959 (water) and 0.00155866 (DMSO) for radical attacks.

11 $$f={\left(\frac{\partial \rho \left(r\right)}{\partial N}\right)}_{v\left(r\right)}$$

12 $$f_{k}^{+}=q_{k}\left(N+1\right)-q_{k}\left(N\right)$$

13 $$f_{k}^{-}=q_{k}\left(N\right)-q_{k}\left(N-1\right)$$

14 $$f_{k}^{0}=\frac{q_{k}\left(N+1\right)-q_{k}\left(N-1\right)}{2}$$

The condensed Fukui functions for nucleophilic attack (*f*_*k*_^+^), electrophilic attack (*f*_*k*_^–^), and radical attack (*f*_*k*_^0^) (eqs 12–14) were computed using Hirshfeld population charge analysis,^56^ where *q*_*k*_(*N* + 1), *q*_*k*_(*N*), and *q*_*k*_(*N* – 1) represent, respectively, the atomic charge for atom *k* in the anionic, neutral, and cationic species of the chalcone. From the condensed Fukui functions, it was calculated the dual descriptor (Δ*f*, eq 15)^59^ and the multiphilic index (Δω, eq 16).^60^ If both Δ*f* and Δω are positive, the reactive site is likely to be susceptible to a nucleophilic attack. Conversely, if both Δ*f* and Δω are negative, the site is more susceptible to an electrophilic attack. Furthermore, the molecular electrostatic potential (MEP) was calculated at the same theoretical level as the Frontier Molecular Orbitals, and the isosurfaces were computed using Gabedit 2.5.0 software^61^ with an isovalue of 0.01.

15 $$\Delta f=f_{k}^{+}-f_{k}^{-}$$

16 Δω=ω·Δf

### 2.8 Molecular Docking

These simulations were carried out following the methodology established by Magalhães et al.^33^ to understand the possible mechanism of action of the CPN4F on the parasite. The three-dimensional structures of the proteins trypanothione reductase (3IUT), cruzain (1K3T), and TcGAPDH (1GXF), classified as oxireductases, were retrieved from the RCSB Protein Data Bank repository (https://www.rcsb.org/). The AutoDockTools program (https://autodocksuite.scripps.edu/adt/) was utilized to remove water residues and cocrystallized ligands. Thereafter, polar hydrogens were added considering the protonation state at physiological pH (pH ∼ 7.4), Gasteiger charges were calculated, and the grid box was computed. The grid box was parametrized according to the following dimensions: 126 × 90 × 126 Å for trypanothione reductase (axes *x* = 44.196, *y* = 3.102, and *z* = −0.054), 116 × 106 × 126 Å for cruzain (axes *x* = 6.612, *y* = −0.436, and *z* = 8.052), and 116 × 112 × 122 Å (axes *x* = 19.822, *y* = 0.455, *z* = 24.804) for TcGAPDH.^62^

The AutoDockVina code (https://vina.scripps.edu/) was configured to perform 50 independent simulations of 20 poses each, using the Lamarckian Genetic Algorithm (LGA), ranked by affinity energy and root mean square deviation (rmsd), based on the methodology of Marinho et al.^63^ Values below 2.0 Å were considered ideal for rmsd,^64^ within an ideal range of affinity energy measured by Gibbs free energy (Δ*G* < −6.0 kcal mol^–1^).^65^

### 2.9 Molecular Dynamics Simulation

To get more accurate results from the molecular docking calculations, molecular dynamics (MD) simulations were used. Using GROMACS 2022.6 software,^66^ we were able to set up the protein to work with the CHARMM36m force field.^67^ The CPN4F ligand was defined using the CHARMM General Force Field (CGenFF) (https://app.cgenff.com/), and then the ligand–protein complex was placed into a box measuring 95*x*, 95*y*, and 95*z* angstroms (Å) and then filled with water molecules (H_2_O) using the transferable intermolecular potential with 3 points (TIP3P) model.^68,69^ The system was made neutral by adding 0.15 mol/L sodium chloride solution, which had 47 sodium (Na^+^) and 34 chloride (Cl^–^) ions in it. The system was then minimized with a steepest descent integrator, configured to stop when the force was <10.0 kJ/mol. After this step, the systems were cooled to a steady temperature, which was done in two steps: the number of particles, the volume, and the temperature are kept constant (*NVT*), and the number of particles, the pressure, and the temperature are kept constant (*NPT*). This meant that the systems could be used for MD production simulations, kept at a constant 310 K and 1.0 bar for 500 ns in triplicate.

The results analysis is based on calculations of something called Root Mean Square Deviation (rmsd). This is worked out by taking the differences between the *x*, *y*, and *z* coordinates of two molecules (1 and 2), squaring these differences (to make sure the result is always positive), adding them together, and dividing by the total number of atoms considered (*N*). The square root of this number is then taken to remove the effect of the power of 2. Here, *N* is the total number of atoms, *r*_*i*2_ is the position of the atom in the last frame that was calculated, and *r*_*i*1_ is the position of the reference atom (the initial position). The average distance between the positions of atoms in two structures can be measured using eq 17.

17 $$rmsd=\sqrt{{\frac{1}{N}\sum_{i=1}^{N}\left(r_{i2}-r_{i1}\right)}^{2}}$$

The root mean square fluctuation (RMSF) is defined as the variation in the position of a given atom, or group of atoms, in relation to the initial structure studied.^70^ The root-mean-square fluctuation (RMSF) was calculated by determining the root-mean-square deviation of the atomic coordinates in relation to the CPN4F-CRUZAIN protein structure over the 500 ns dynamics time, allowing for the evaluation of the flexibility or rigidity of different regions of the protein, with a particular emphasis on highlighting the parts that present greater fluctuations or conformational variations. In this context, *N* signifies the total number of simulation frames, *r*_*i*_(*t*) denotes the position of the atom at time *t*, and *r*_*i*_ signifies the average position of atom *i* in the *N* simulated frames (eq 18)­

18 $$rmsd=\sqrt{{\frac{1}{N}\sum_{i=1}^{N}\left(r_{i}\left(t\right)-r_{i}\right)}^{2}}$$

Equation 19 describes Δ*G*_bind_, which represents the free energy of binding between the components of the system. The terms *E*_vdW_ and *E*_ele_ correspond to the van der Waals interactions and electrostatic energies. The *G*_GB_ component refers to the polar contributions to the solvation energy, while *G*_SA_ represents the nonpolar contributions. Finally, the term *T*Δ*S* is the product of the absolute temperature (*T*) and the entropy change (Δ*S*), estimated through the analysis of normal modes of the vibrational frequencies^71,72^ (eq 19).

19 $$\Delta G_{bind}=E_{vdW}-E_{ele}+G_{GB}+G_{SA}-T\Delta S$$

### 2.10 MPO-Based ADME and Toxicity Prediction

Initially, the CPN4F Simplified Molecular Input Line Entry System (SMILES) linear code was subjected to a drug likeness test based on the Lipinski “rule of five” criteria: partition coefficient (log *P*) ≤ 5, molecular weight (MW) ≤ 500 g/mol, H-bond donor (HBD) ≤ 5, H-bond acceptor (HBD) ≤ 10;^73^ and the Ghose rule: −0.4 < log *P* ≤ 5.6, 160 < MW ≤ 480 g/mol, 40 < molar refractivity (MR) ≤ 130, 20 < atoms ≤ 70,^74^ using the SwissADME server (http://www.swissadme.ch/), where the results were conditioned to multidimensional analysis using the chemical intelligence software DataWarrior, OpenChemLib (https://www.openmolecules.org/datawarrior/). This was then compared with the enzyme inhibitors of the *T. cruzi* proliferative cycle (BZN, KB2, BRZ, QUM, and nifurtimox).

The two-dimensional representation of the chemical structure of CPN4F was plotted in the academic software MarvinSketch version Iodine 7, Chemaxon (https://chemaxon.com/marvin), and optimized by the Merck Molecular Force Field 94 (MMFF94) classical mechanics force field to generate the molecular lipophilicity potential (MLP) map, as shown in eq 20

20 $${MLP}_{k}=\sum_{i=1}^{N}F_{i}\cdot f\left(d_{ik}\right)$$

where each fragment *i* is assigned a lipophilicity value *F*, where *N* is the number of molecular fragments. In addition, the spatial distance function *k*, which measures the proximity between each fragment *i*, generates a surface map that varies from shades of red (for hydrophilic regions) to shades of blue (for lipophilic regions). The results were associated with the prediction of lipophilicity descriptors, which include the partition coefficient (log *P*), distribution coefficient (log *D*), acid ionization constant (p*K*_a_), and pH-dependent net charge distribution. The MLP was generated using Python Molecular Graphics software.^75^

The ligand was subjected to quantitative drug likeness prediction by the multiparameter optimization (MPO) system from Pfizer, Inc., as shown in eq 21

21 $$d=\sum_{i=1}^{n}w_{k}T_{k}\left(x_{k}^{0}\right)$$

where the desirability function (*d*) is determined by the weighting factor (*w*) of each calculated attribute (*k*), taking into account whether the calculated value (*x*) is inside (*x* ≤ *x*_a_) or outside (*x*_b_ ≤ *x*) the ideal limits (*T*(*x*)). These attributes include log *P* ≤ 3, log *D* ≤ 2, MW ≤ 360 g/mol, a Topological Polar Surface Area (TPSA) of 40 to 90 Å^2^, HBD ≤1, and p*K*_a_ of the most basic center ≤8 (*n* = 6).^76^ This evaluation results in a score ranging from 0 (low pharmacokinetic viability) to 6 (high pharmacokinetic viability), directly associated with the alignment between permeability and metabolic stability, estimated by the lipophilicity-metabolism efficiency (LipMetE) descriptor, as shown in eq 19 ^77^

22 $$LipMetE=\log D_{pH7.4}-\log CL$$

where log *D* is the lipophilicity estimated at physiological pH (pH 7.4) and log CL is the logarithm of hepatic clearance at −log_10_(mL/min/kg), resulting in a score inversely proportional to the metabolic stability of the compound (ideal value between 0 and 2.5).

The results were related to the prediction of absorption, distribution, metabolism, and excretion (ADME) descriptors from the ADMETlab 2.0 (https://admetmesh.scbdd.com/) and pkCSM (https://biosig.lab.uq.edu.au/pkcsm/) servers, which include apparent permeability (*P*_app,A→B_), P-glycoprotein (P-gp) substrate, oral bioavailability fraction (*F* %), unbound fraction in blood plasma (Fu), activity in the central nervous system (CNS), and intrinsic hepatic clearance rate (CL_int,u_).

The SOMP servers—Way2Drug (http://www.way2drug.com/somp/), XenoSite (https://xenosite.org/), and STopTox (https://stoptox.mml.unc.edu/)—and the identification of structural alerts from SureChEMBL^78^ were used to relate the results of the LipMetE analysis to the prediction of the site of metabolism. The graphical response of these servers indicates the sensitivity of molecular functional groups to being metabolized by different cytochrome P450 (CYP450) isoforms, as well as whether they can cause liver damage (DILI descriptor) or be mutagenic.^79^ Finally, the LD_50_ in rats was estimated for the compounds using a structural similarity model with pharmacophores deposited in the ProTox-3.0 server database (https://tox.charite.de/protox3/), as a method of estimating acute toxicity in humans.

## 3 Results and Discussion

### 3.1 Structure Properties

#### 3.1.1 Structural Determination from Quantum Chemical Calculations

Quantum chemical calculations were performed using the density functional theory (DFT) method at the B3LYP/6-311++G­(d,p) computational level to optimize the structure of the CPN4F chalcone for minimum energy. The calculations were conducted in two different chemical environments: water and DMSO, which were implemented as implicit solvents. These environments were chosen, respectively, based on their relevance to human physiological composition as a possible new drug, its properties as a protic solvent, and the solubility of the title chalcone in organic polar and aprotic solvents.

The optimized structures of the CPN4F chalcone in water (CPN4F-W) and DMSO (CPN4F-D) are shown in Figure 2. The chemical structure of the molecule, regardless of the solvent, as shown in Figure 2, consists of two aromatic rings: ring A on the left and ring B on the right. It includes a carbonyl group, two olefinic carbons that are connected by a double bond, designated as the alpha (Cα) and beta (Cβ) carbons, respectively. Additionally, the structure contains three methoxyl groups, a hydroxyl group attached in the ortho position of ring A, and a fluorine atom bonded in the para position of ring B. Regardless of the two types of solvents used, all methoxyl groups were found to be positioned above or below the plane of ring A, with angles ranging from 116° (C1C2O10) to 124° (C1C6O9). The hydroxyl group in ring A also exhibited a slight distortion in the C3C4O11 angle, measuring 121.543° in water and 121.552° in DMSO, resulting in a modulus difference of 0.009°. This variation in the angle leads to a slight difference in the length of the intramolecular hydrogen bond between the hydrogen atom (H12) of the hydroxyl group and the oxygen atom (O26) of the carbonyl group: 1.59217 Å in water and 1.59330 Å in DMSO, with a modulus difference of 0.0013 Å. Although this difference is small, it suggests that the intramolecular hydrogen bond in the water-optimized CPN4F chalcone is shorter and likely stronger. This may indicate a subtle change in the reactivity of the compound in protic media.

**Figure 2:**
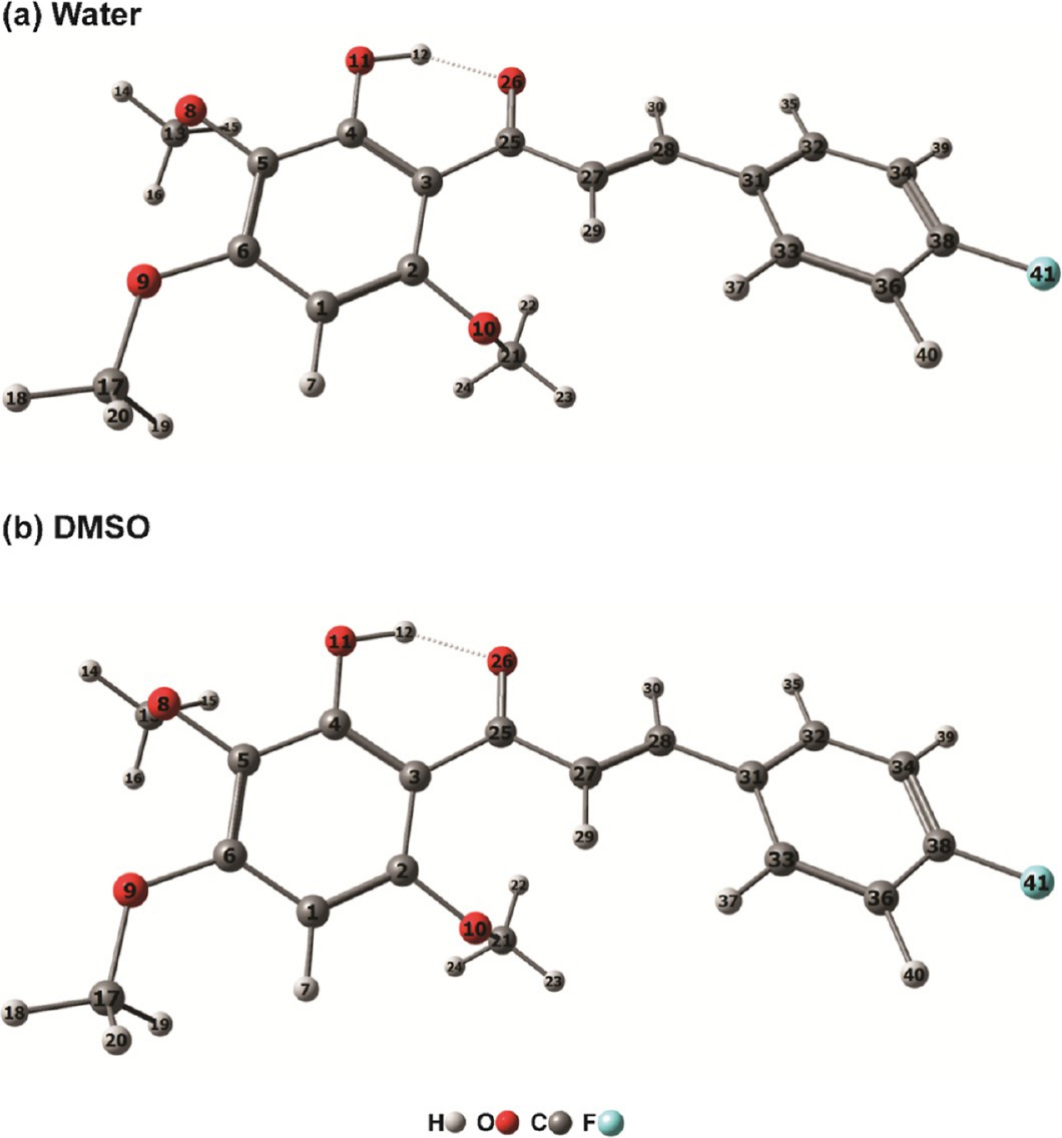
Molecular structure of the chalcone (*E*)-3-(4-fluorophenyl)-1-(2-hydroxy-3,4,6-trimethoxyphenyl)­prop-2-en-1-one (CPN4F) in (a) water and (b) DMSO environments as implicit solvents obtained at the B3LYP/6-311++G­(d,p) level of theory.

The main carbon skeleton of the molecular structure does not lie entirely in the same plane. For ring A, the dihedral angle C2C3C25C27 deviates from the main plane by approximately 15.078° for the CPN4F-W variant and 15.155° for CPN4F-D, resulting in a modulus difference of 0.077°. This difference may be significant for the biological behavior of the molecule when it acts as a drug targeting proteins in the bloodstream under physiological conditions. In contrast, ring B is almost completely coplanar with the α,β-unsaturated carbonyl structure. The dihedral angle C27C28C31C33 measures about 0.325° for CPN4F-W and 0.147° for CPN4F-D, leading to a modulus difference of 0.092°. Additionally, the dihedral angle C27C28C31C32 is approximately 179.100° for CPN4F-W and 179.009° for CPN4F-D, with a modulus difference of 0.091°. This can be interpreted as a minor adjustment in the overall molecular structure caused by hydrogen bonding, which is shorter in water compared to DMSO. The entire structure adapts to this change to maintain electronic delocalization stemming from resonance effects.

The α,β-unsaturated carbonyl structure remains nearly identical when comparing the two molecular geometries in water and DMSO. The dihedral angle C25C27C28C31 measures approximately 179.155° for CPN4F-W in water and 179.160° for CPN4F-D in DMSO, resulting in a negligible difference of just 0.005°. This indicates that the core structure of the chalcone CPN4F does not significantly change with the type of solvent, either protic or aprotic. However, the ligands may rearrange themselves to ensure maximum delocalization of electronic density, as well as to achieve a geometrically optimal configuration for interacting with other compounds or biological macromolecules.

Finally, the presence of the fluorine atom (F41) bonded at the para-position in ring B shows minimal differences in its dihedral angles. For the CPN4F-W structure, the dihedral angle F41C38C34C32 is approximately 179.733°, while for the CPN4F-D structure, it is about 179.728°, resulting in a difference of only 0.005°. Similarly, the dihedral angle F41C38C36C33 measures around 179.772° for CPN4F-W and 179.765° for CPN4F-D, yielding a difference of 0.007°. These results are expected due to the nature of the ligand attached to the aromatic ring: the small fluorine atom. This tiny atom does not introduce any significant steric effects when the chalcone interacts with other molecules, as ring B is used to make these interactions.

#### 3.1.2 NMR Analysis

The ^1^H NMR spectrum (shown in Figure 3, with an expanded view in Figure 4) reveals a signal at 13.93 ppm corresponding to the hydrogen of the hydroxyl (OH) group. Additionally, there are three signals at 3.96 ppm (two of them) and 3.84 ppm, which are attributed to the methoxyl hydrogens. At 7.82 ppm (*J* = 15.6 Hz) and 7.83 ppm (*J* = 15.6 Hz), two doublets are observed, corresponding to the α,β-unsaturated hydrogens. The coupling constants (*J*) confirm that the double bond has E stereochemistry. A singlet observed at 6.07 ppm is associated with the hydrogen bonded to carbon 3′ of ring A. Finally, the signals at 7.63 ppm (H-2 and H-6, *J* = 8.58 Hz) and 7.65 ppm (H-3 and H-5, *J* = 8.58 Hz) pertain to the aromatic hydrogens of ring B.

**Figure 3:**
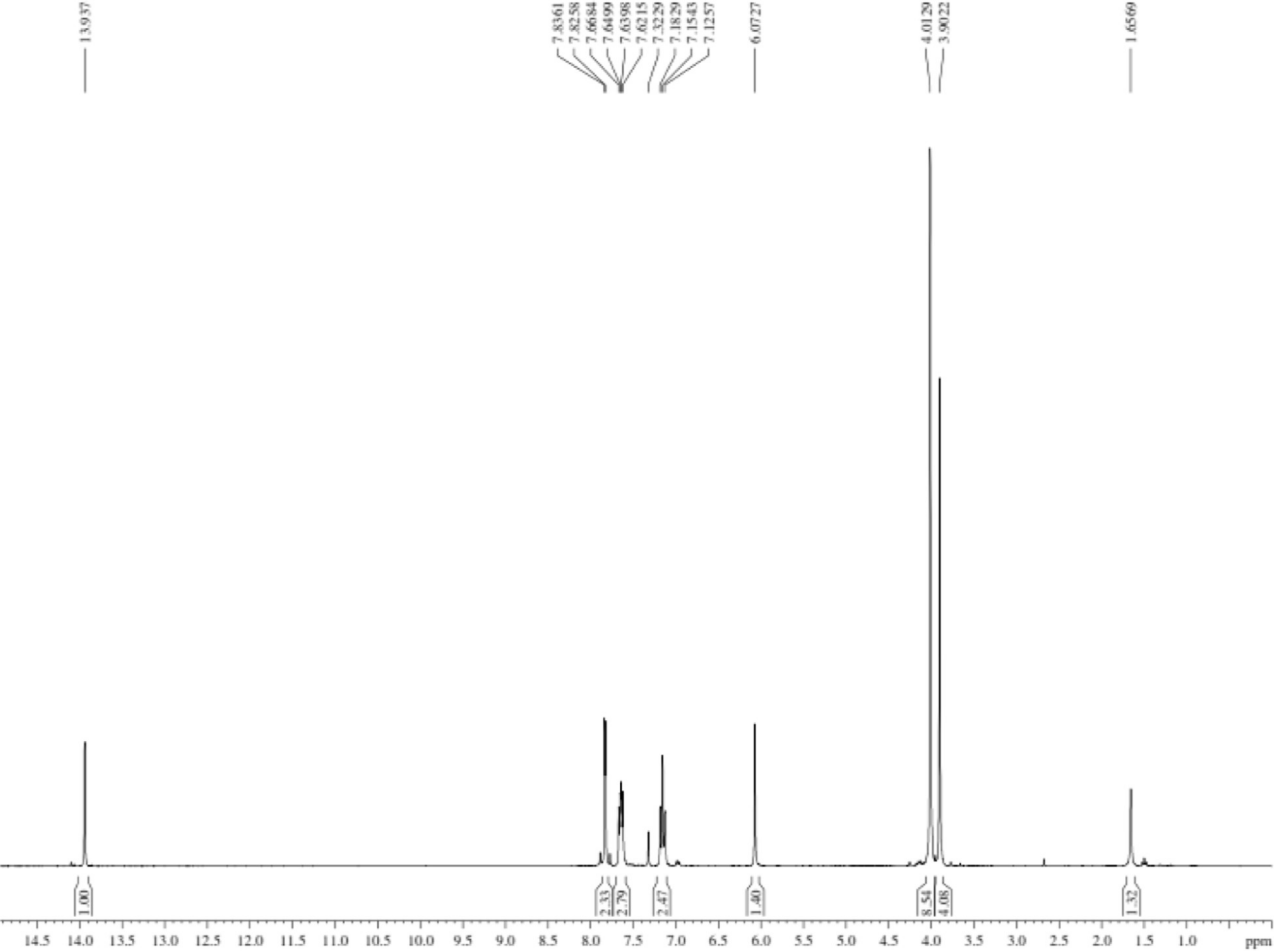
^1^H NMR spectrum (CDCl_3_, 500 MHz) for chalcone (*E*)-3-(4-fluorophenyl)-1-(2-hydroxy-3,4,6-trimethoxyphenyl)­prop-2-en-1-one (CPN4F).

**Figure 4:**
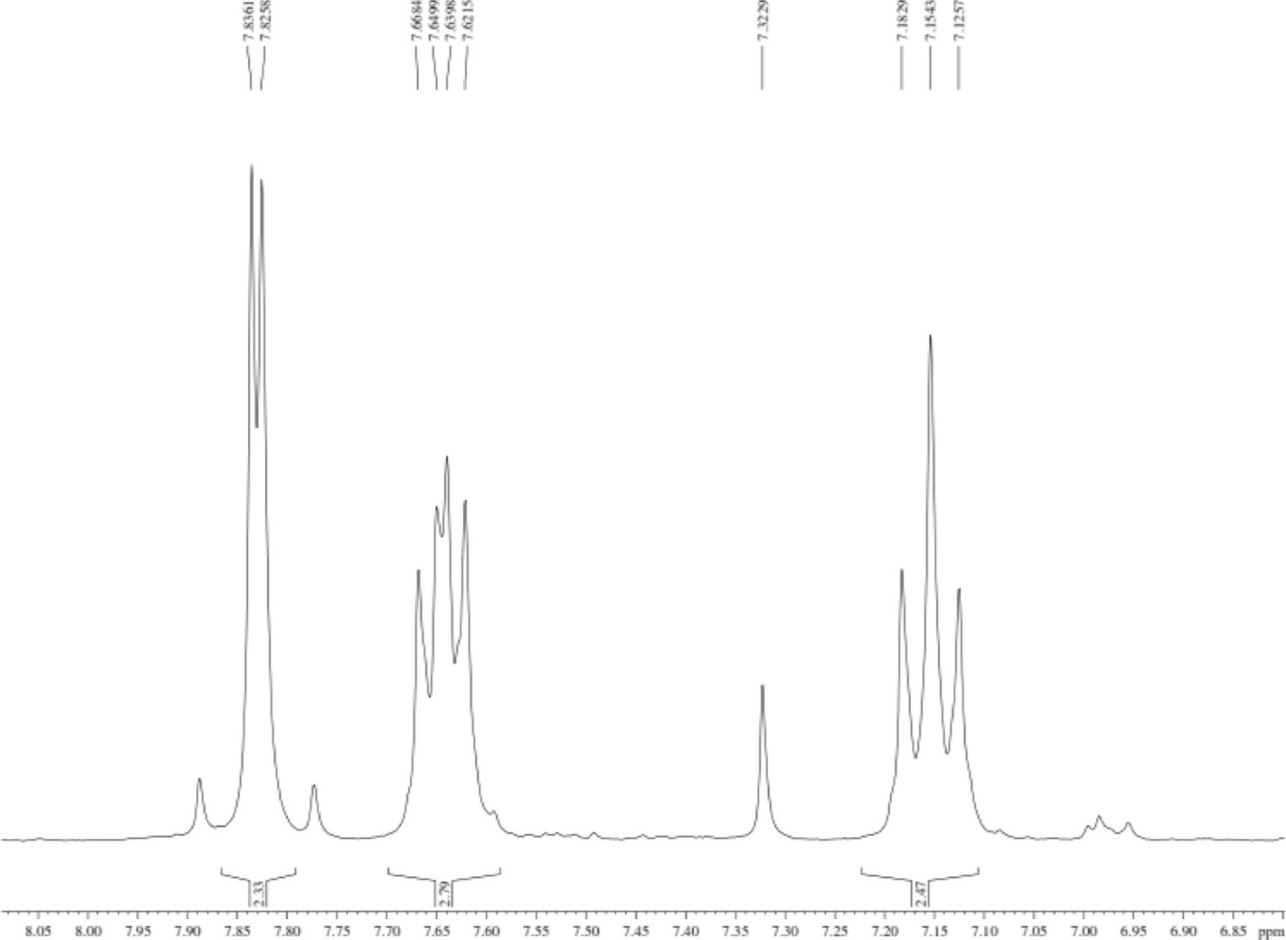
Expansion of the ^1^H NMR spectrum (CDCl_3_, 500 MHz) of chalcone (*E*)-3-(4-fluorophenyl)-1-(2-hydroxy-3,4,6-trimethoxyphenyl)­prop-2-en-1-one (CPN4F).

The ^13^C NMR spectrum (shown in Figure 5 and extended in Figure 6) reveals a signal for the α,β-unsaturated carbonyl at 193.2 ppm. The ketone shows an absorption at 203.8 ppm. However, the presence of the α,β unsaturation results in a shift to a lower field. This shift is likely due to charge delocalized at the benzene ring or the double bond, which reduces the electron deficiency of the carbonyl group. The olefinic carbons at the α and β positions appear at 141.5 and 127.4 ppm, respectively. Signals at 60.9, 56.3, and 56.2 ppm correspond to the methoxyl carbons. For ring A, the signals at 159.6 ppm (C-4′), 158.7 ppm (C-6′ and C-2′), 130.4 ppm (C-5′), 107.1 ppm (C-1′), and 87.4 ppm (C-3′) represent the carbons. Meanwhile, the carbons in ring B (C-1 to C-6) can be observed at 131.2 ppm (C-1), 131.9 ppm (C-2/6), 131.9 ppm (C-3/5), and 141.5 ppm (C-4) as detailed in Table 1.

**Figure 5:**
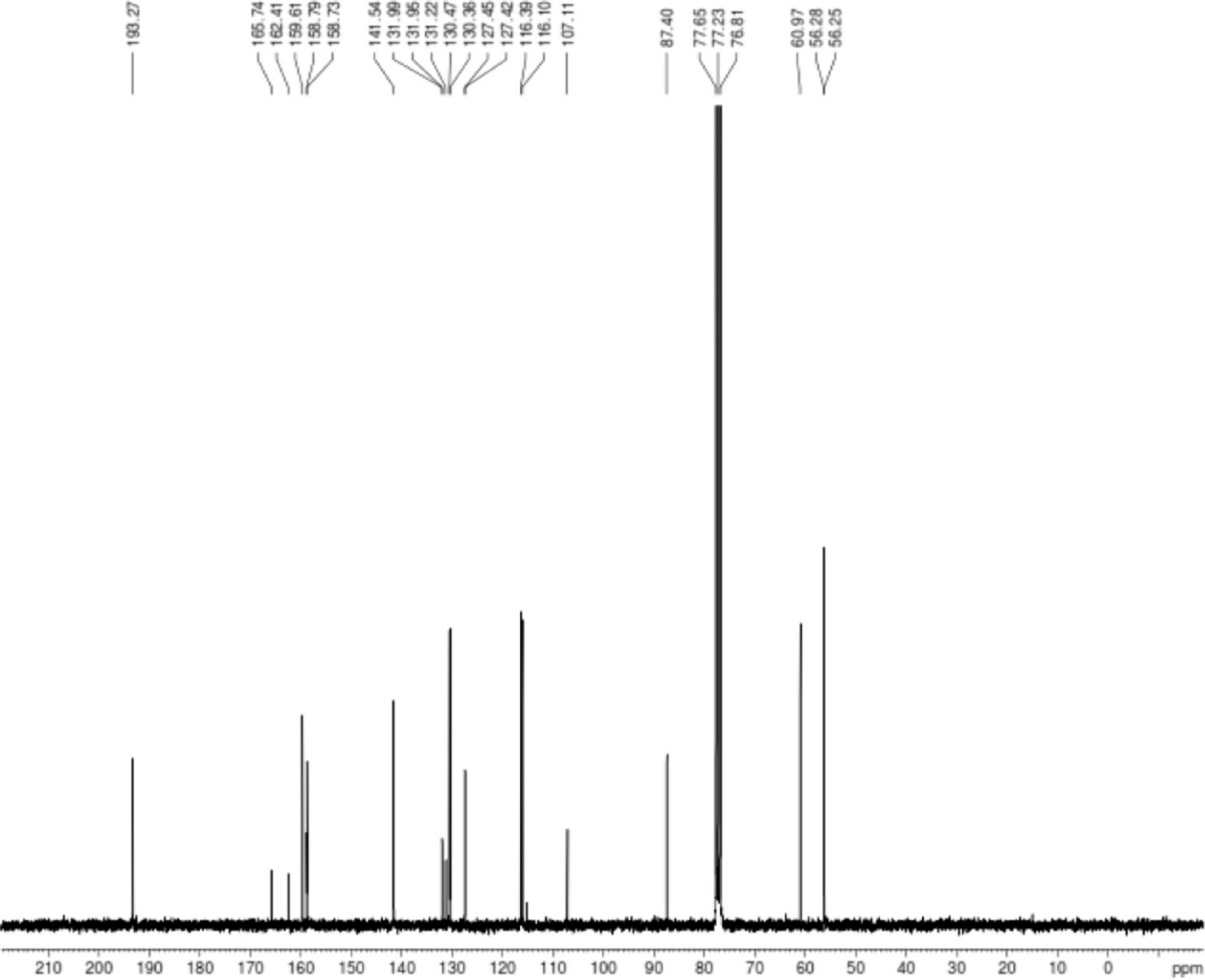
^13^C NMR spectrum (CDCl_3_, 125 MHz) of chalcone (*E*)-3-(4-fluorophenyl)-1-(2-hydroxy-3,4,6-trimethoxyphenyl)­prop-2-en-1-one (CPN4F).

**Figure 6:**
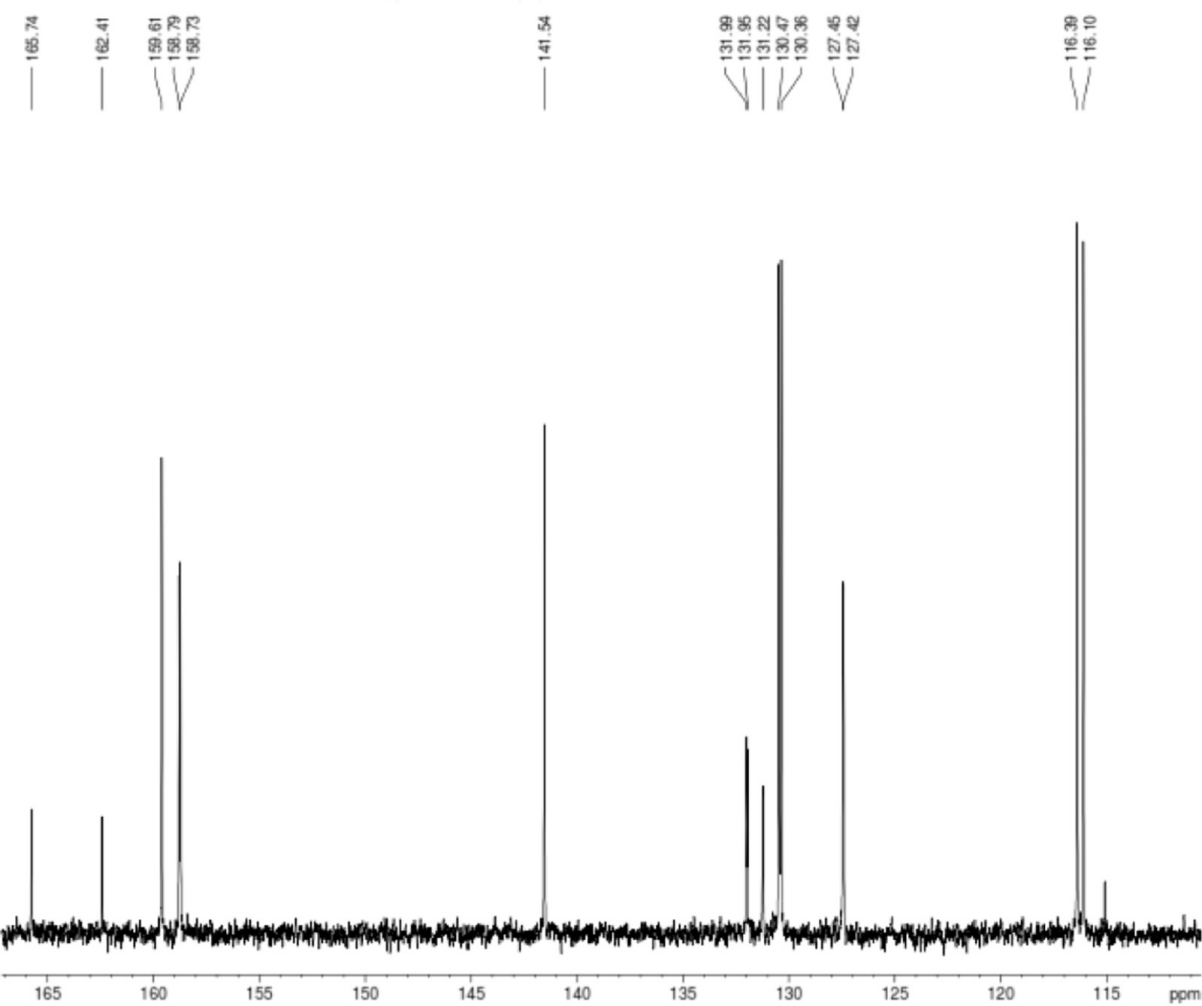
Expansion of the ^13^C NMR spectrum (CDCl_3_, 125 MHz) of chalcone (*E*)-3-(4-fluorophenyl)-1-(2-hydroxy-3,4,6-trimethoxyphenyl)­prop-2-en-1-one (CPN4F).

**Table 1:** ^1^H and ^13^C NMR Experimental Data of the Chalcone (*E*)-3-(4-Fluorophenyl)-1-(2-hydroxy-3,4,6-trimethoxyphenyl)­prop-2-en-1-one (CPN4F) in CCDl_3_ ^a^

carbon atom	δ_C(exp)_	hydrogen atom	δ_H(exp)_
C3	107.1		
C4	158.7		
C5	130.4		
C6	159.6		
C1	87.4	H7	6.07 (s)
C2	158.7		
C13	60.9	H14, H15, H16	3.96 (s)
C17	56.3	H18, H19, H20	3.96 (s)
C21	56.2	H22, H23, H24	3.84 (s)
C25	193.2		
C31	131.9		
C32/C33	131.2	H35 and H37	7.63 (d, *J* = 8.58 Hz)
C34/C36	131.9	H39 and H40	7.65 (d, *J* = 8.58 Hz)
C38	141.5		
C27 (C_α_)	127.4	H29	7.82 (d, *J* = 15.60 Hz)
C28 (C_β_)	141.5	H30	7.83 (d, *J* = 15.60 Hz)

a The chemical shifts in δ_C_ and δ_H_ are in ppm.

To validate the quantum chemical calculations, the isotropic shielding values for both ^1^H and ^13^C NMR were computed in the same deuterated solvent (CDCl_3_) as used in the experiments, utilizing the B3LYP/6-311++G­(d,p) computational level. The results for the chalcone optimized in water (CPN4F-W) and DMSO (CPN4F-D) are presented in Table 2. The results show that the theoretical chemical shift values remained relatively unchanged when chalcone was optimized in either water or DMSO. This indicates that despite some minor structural differences, these variations were not significant enough to affect the data observed in the NMR measurement data. To understand the relationship between the experimental and theoretical values, linear regression analysis was performed for both of the media (water and DMSO). The results of this analysis are illustrated in Figure 7 (for water) and Figure 8 (for DMSO). The coefficient of determination (*R* ^2^) for both water and DMSO is 0.99022, with linear equations of *y* = 0.93196*x* + 0.38831 (water) and *y* = 0.93199*x* + 0.39068 (DMSO). These results indicate a strong correlation between the experimental and theoretical data. Therefore, quantum chemical calculations can be effectively utilized to characterize the electronic and reactivity properties of the chalcone CPN4F.

**Table 2:** ^1^H and ^13^C NMR Theoretical Data of the Chalcone (*E*)-3-(4-Fluorophenyl)-1-(2-hydroxy-3,4,6-trimethoxyphenyl)­prop-2-en-1-one (CPN4F) in CCDl_3_ ^a^

carbon atom	δ_C(CPN4F‑W)_	δ_C(CPN4F‑D)_	hydrogen atom	δ_H(CPN4F‑W)_	δ_H(CPN4F‑D)_
C3	117.3	117.3	H7	6.4	6.4
C4	169.4	169.4	H12	13.9	13.9
C5	141.4	141.4	H14	4.0	3.9
C6	169.1	169.1	H15	3.7	3.7
C1	104.0	104.0	H16	3.9	3.9
C2	170.3	170.3	H18	4.3	4.3
C13	63.6	63.5	H19	4.0	4.0
C17	59.9	59.8	H20	3.9	3.9
C21	68.7	68.7	H22	3.6	3.6
C25	200.4	200.4	H23	4.1	4.1
C31	140.1	140.1	H24	3.5	3.5
C32	143.9	143.9	H29	8.6	8.6
C33	135.4	135.4	H30	8.3	8.3
C34	123.1	123.1	H35	7.9	7.9
C36	123.6	123.6	H37	8.5	8.5
C38	176.7	176.7	H39	7.5	7.5
C27 (C_α_)	131.3	131.3	H40	7.5	7.5
C28 (C_β_)	152.8	152.8	-	-	-

a The theoretical chemical shifts in δ_C_ and δ_H_ are in ppm.

**Figure 7:**
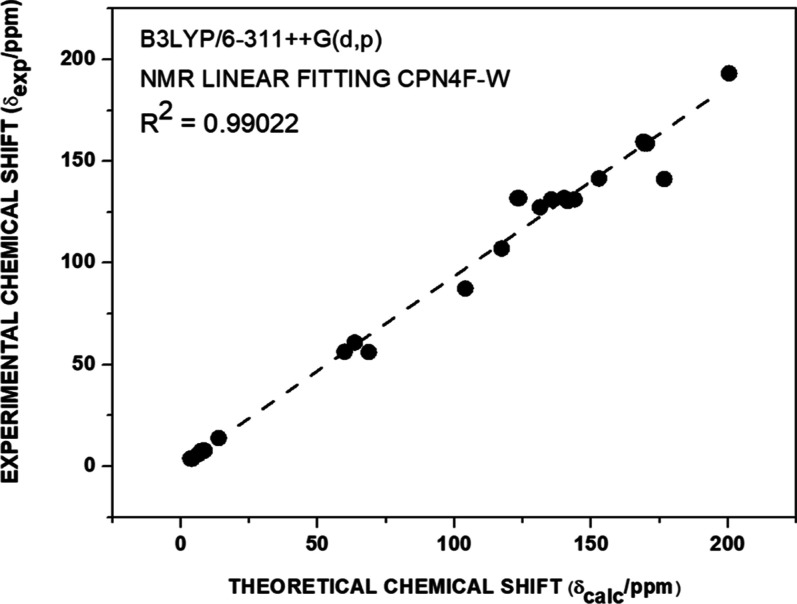
Linear fitting between the experimental and theoretical values of the isotropic chemical shifts for hydrogen and carbon atoms for CPN4F-W chalcone.

**Figure 8:**
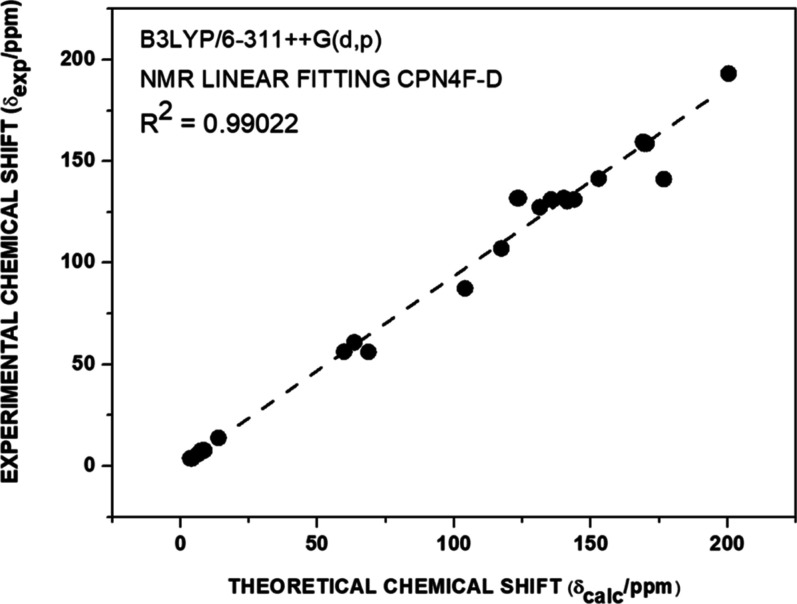
Linear fitting between the experimental and theoretical values of the isotropic chemical shifts for hydrogen and carbon atoms for CPN4F-D chalcone.

#### 3.1.3 IR Analysis

The FT-IR spectrum of the chalcone CPN4F is presented in Figure 9. It shows a total of 20 vibrational modes. Although experimental spectra have been obtained, theoretical calculations are essential not only to validate the reliability of the DFT method employed but also to aid in the detailed assignment of vibrational modes and provide a consistent basis for characterizing the electronic and reactivity properties of chalcone, expanding the scope of experimental analyses. In this sense, to identify each fundamental vibrational mode, quantum chemical calculations were conducted at the B3LYP/6-311G­(d,p) level of theory, both in aqueous environments and in DMSO for the chalcone CPN4F. The results of these calculations are detailed in Table 3. To validate the calculations, linear fitting was performed for both the water and DMSO environments, with the findings illustrated in Figure 9 (for water) and Figure 10 (for DMSO). The coefficients of determination were determined to be 0.99867 for water, with a fitting equation of *y* = 0.99813*x* – 0.89933, and 0.99949 for DMSO, with the equation *y* = 1.01028*x* – 16.37164. The wavenumbers show only slight variations when the solvent is changed. This finding aligns with the observations made in the NMR section. Although there are minor structural differences, they are not significant enough to alter the spectroscopic properties of the title chalcone.

**Figure 9:**
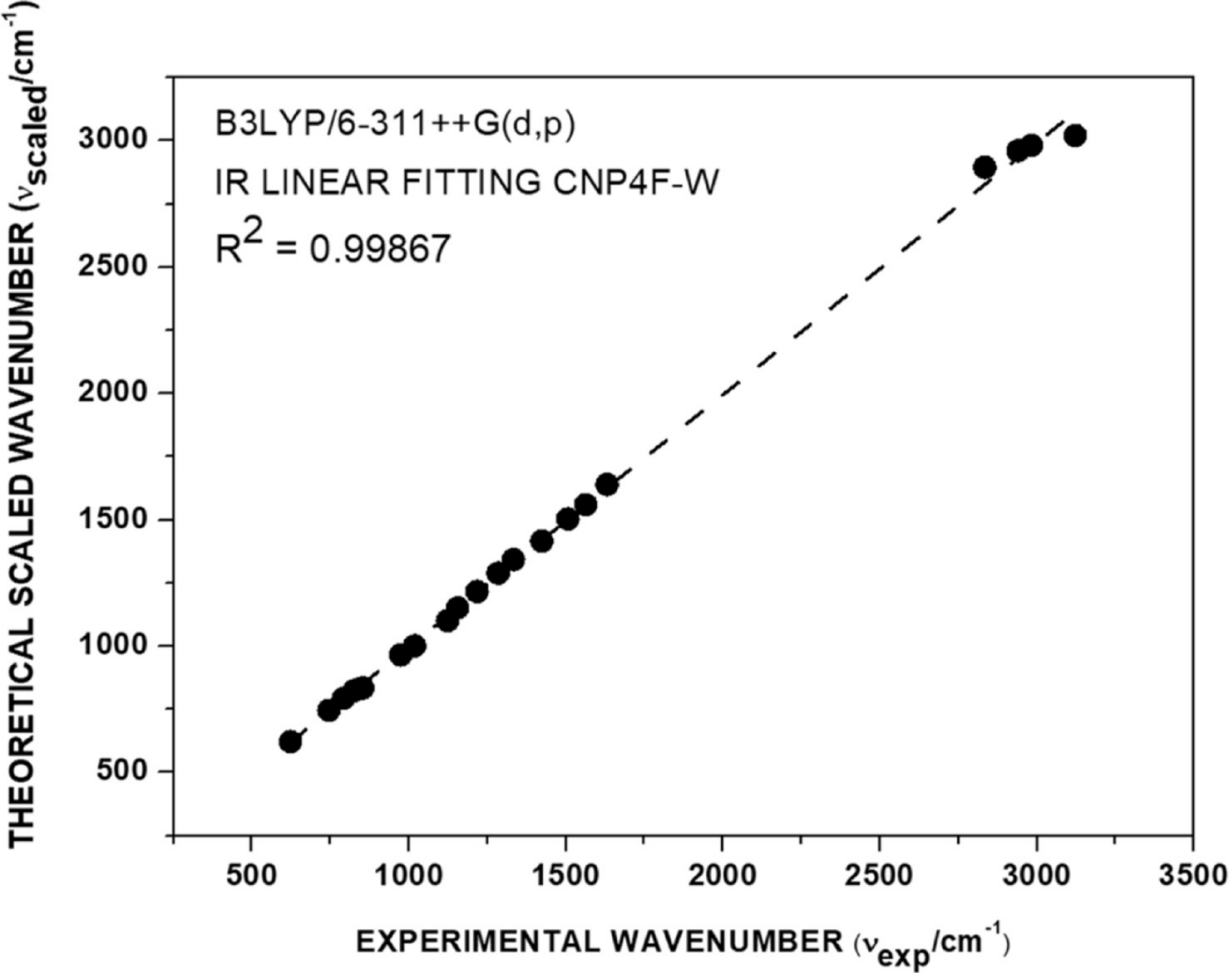
Linear fitting between the experimental and theoretical values of the wavenumbers of fundamental vibrational modes for CPN4F-W chalcone.

**Table 3:** Experimental and Theoretical Data of Wavenumbers for the Fundamental Vibrational Modes of the Chalcone (*E*)-3-(4-Fluorophenyl)-1-(2-hydroxy-3,4,6-trimethoxyphenyl)­prop-2-en-1-one (CPN4F) in Both Water and DMSO Environments

experimental wavenumber (cm^–1^)	scaled theoretical wavenumber (cm^–1^) for CPN4F-W	scaled theoretical wavenumber (cm^–1^) for CPN4F-D
623	623	623
745	747	747
792	793	794
825	824	825
853	835	835
974	965	965
1018	1002	1002
1123	1101	1101
1155	1151	1146
1217	1217	1217
1284	1289	1289
1333	1343	1343
1423	1416	1417
1506	1504	1504
1563	1560	1561
1631	1641	1640
2834	2895	2895
2940	2961	2973
2983	2982	2985
3122	3102	3102

**Figure 10:**
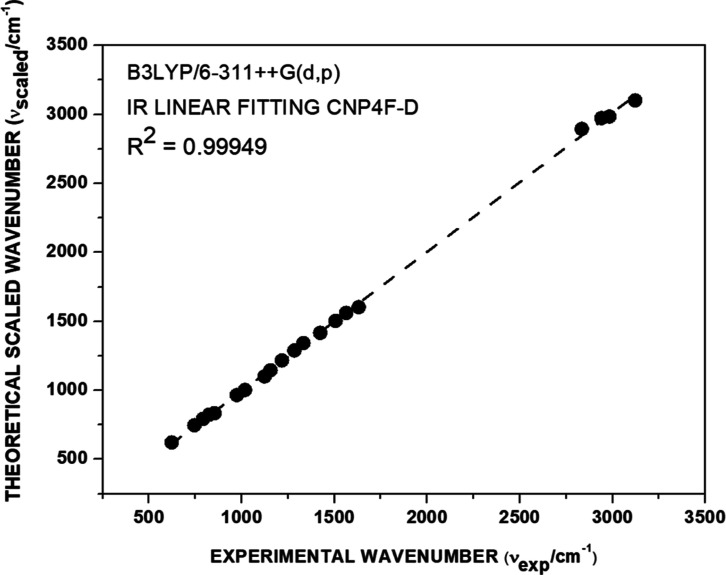
Linear fitting between the experimental and theoretical values of the wavenumbers of fundamental vibrational modes for CPN4F-D chalcone.

#### 3.1.4 Frontier Molecular Orbitals

The frontier molecular orbitals, the HOMO and the LUMO, obtained at the B3LYP/6-311++G­(d,p) level of theory in both water and DMSO environments are shown in Figure 11. Figure 11 illustrates that the isosurface distributions of both the HOMO and LUMO are nearly identical for the two solvents used in the optimization. However, there is a noticeable difference in the wave function signals for certain atoms; the LUMO exhibits phase swaps when comparing water to DMSO. The square of the wave function of the orbitals determines the spread of the electron density. Therefore, the signal alteration did not lead to different results.

**Figure 11:**
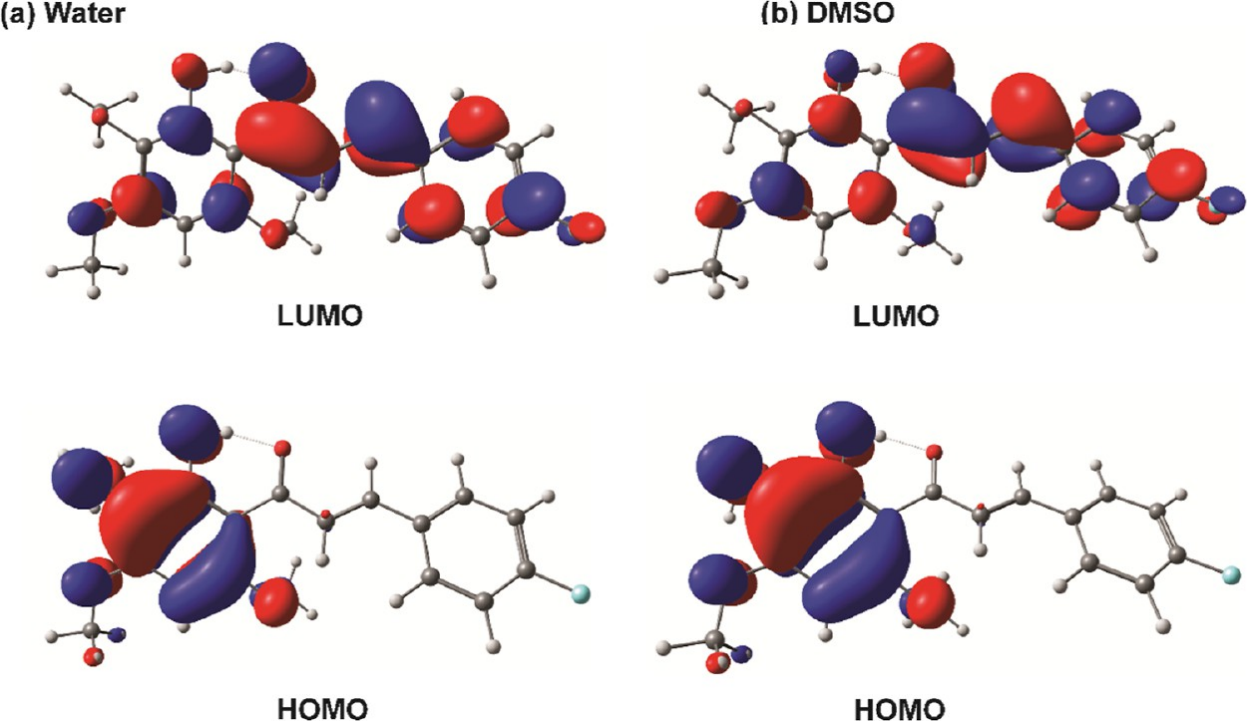
Frontier Molecular Orbitals (HOMO and LUMO) for the chalcone CPN4F in both (a) water and (b) DMSO environments obtained at the B3LYP/6-311++G­(d,p) computational level.

In this context, it is evident that the HOMO for both water and DMSO is predominantly distributed over the aromatic ring A and the three methoxy groups, or at the atoms C1, C2, C3, C4, C5, C6, O8, O9, O10, and O11. The results indicate that the chalcone has a unique electron donor region. The presence of methoxy groups increases the electron density in ring A due to a mesomeric effect, making it richer in electron density and more available for interaction with other molecules, acting as a nucleophile. Furthermore, the π-bonding characteristics of the HOMO are significant; its isosurfaces align along the internuclear axes and extend above and below the plane of the ring.

In the case of the LUMO, it is distributed across various parts of the molecular structure. Specifically, it can be found on aromatic ring A, the methoxy groups (O9C17 and O10C21), the α,β-unsaturated structure, aromatic ring B, and the fluorine atom, or at the atoms C2, C4, C6, O9, O10, O11, C25, O26, C28, C33, C32, C38, and F41. Notably, the LUMO exhibits π-antibonding characteristics, with its isosurfaces extending into areas outside the internuclear axis, as well as both above and below the plane of the molecule. The LUMO is associated with the electron density acceptor character of the molecule. This chalcone can interact with other compounds by accepting electron density from various regions of its molecular structure due to the distribution of the LUMO. It is important to note that the region of ring B containing the fluorine atom is likely to be more susceptible to nucleophilic attack. This is because the extra electron density can gather near the highly electronegative fluorine atom, allowing it to be dispersed through resonance in ring B and the α,β-unsaturated structure. Since ring B is coplanar with it, while ring A deviates from the main plane of the molecule, this deviation may reduce the overlap of the orbitals and weaken the resonance effect.

#### 3.1.5 Global Quantum Reactivity Chemical Descriptors

The global quantum reactivity descriptors were computed for both water and DMSO environments from the energy values of the HOMO and LUMO, and the results are shown in Table 4.

**Table 4:** Global Quantum Reactivity Descriptors Calculated at the B3LYP/6-311++G­(d,p) Level of Theory for Water (CPN4F-W) and DMSO (CPN4F-D) Environments

global quantum reactivity descriptors	CPN4F-W	CPN4F-D
HOMO energy (*E*_HOMO_/eV)	–6.313	–6.311
LUMO energy (*E*_LUMO_/eV)	–2.566	–2.564
energy gap (Δ*E*_Gap_/eV)	3.748	3.747
ionization potential (*I*/eV)	6.313	6.311
electron affinity (A/eV)	2.566	2.564
electronegativity (χ/eV)	4.440	4.438
global hardness (η/eV)	1.874	1.873
global softness (σ/eV^–1^)	0.534	0.534
electrophilicity index (ε/eV)	5.259	5.256
nucleophilicity index (ω/eV^–1^)	0.190	0.190

From Table 4, it is evident that the HOMO energy values are nearly identical, with only a slight variation of 0.002 eV. This result indicates that chalcone is likely to exhibit similar behavior in terms of donor power regardless of the solvent type, whether protic or aprotic. This expectation aligns with the HOMO distribution analysis, which suggests that chalcone should function similarly to a nucleophile in any solvent. The LUMO energy behaves similarly in different solvents (water and DMSO) with a difference of about 0.002 eV. This suggests that chalcone may function as a solvent-independent electrophile. This observation aligns with the findings regarding the LUMO distribution as both solvents exhibit identical electron density distributions. This indicates that the electronic properties of chalcone remain unaffected by whether the solvent is protic or aprotic.

When comparing all the descriptors, it is evident that they present nearly identical values, with the largest difference observed in the electrophilicity index, which is approximately 0.003 eV, a relatively small difference. These descriptors indicate that this chalcone is likely to exhibit a greater electron density acceptor character than its electron density donor capability. This is evidenced by the electrophilicity index being higher than the nucleophilicity index, as well as the global hardness exceeding the global softness. As a result, this molecule tends to maintain a more rigid electron density and is less prone to donating electrons. However, due to the resonance effect dispersed throughout its molecular structure, along with the presence of an electronegative fluorine atom, this molecule can effectively accept and accommodate an extra negative charge, making it an excellent electrophile. Furthermore, its properties remain consistent regardless of the type of solvent it is in, whether protic or aprotic. Moreover, the primary interactions of this chalcone with other molecules, particularly with the amino acids of target proteins, are π interactions and hydrogen bonds.

#### 3.1.6 Fukui Functions and the Molecular Electrostatic Potential

To continue the electronic characterization of the chalcone in water and DMSO environments, the electronic and condensed Fukui functions and the Molecular Electrostatic Potential (MEP) were computed for the chalcone (CPN4F-W and CPN4F-D). The electronic Fukui functions for the nucleophilic (*f* ^+^), electrophilic (*f* ^–^), and radical attack (*f* ^0^) are shown in Figure 12. The analysis shows that, regardless of the solvent used, the distributions of the three electronic Fukui functions are quite similar. This observation aligns with previous findings in the analysis of the Frontier Molecular Orbitals (FMO) and global reactivity descriptors, leading to the conclusion that the electronic properties of this chalcone are independent of the medium. In terms of nucleophilic attack, the chalcone is expected to act as an electrophile, with the most susceptible atoms being C2, C4, C6, O9, O10, O11, C25, O26, C28, C32, C33, C38, and F41. Notably, these atoms correspond to those where the LUMO is mainly distributed; these atoms are the most susceptible to acting as electrophiles during a nucleophilic attack. In the case of electrophilic attack, chalcone acts as a nucleophile. According to the results shown in Figure 12, the most likely atoms involved are C1, C2, C3, C4, C5, C6, O8, O9, O10, and O11. These atoms correspond to the regions where the HOMO is distributed, indicating that they are the most probable sites for nucleophilic activity during an electrophilic attack. For radical attack, the most susceptible atoms are C1, C2, C4, C5, C6, O8, O9, O10, O11, C25, O26, C28, C32, C33, and C38. Almost the entire molecular structure of this chalcone can be subjected to radical attack.

**Figure 12:**
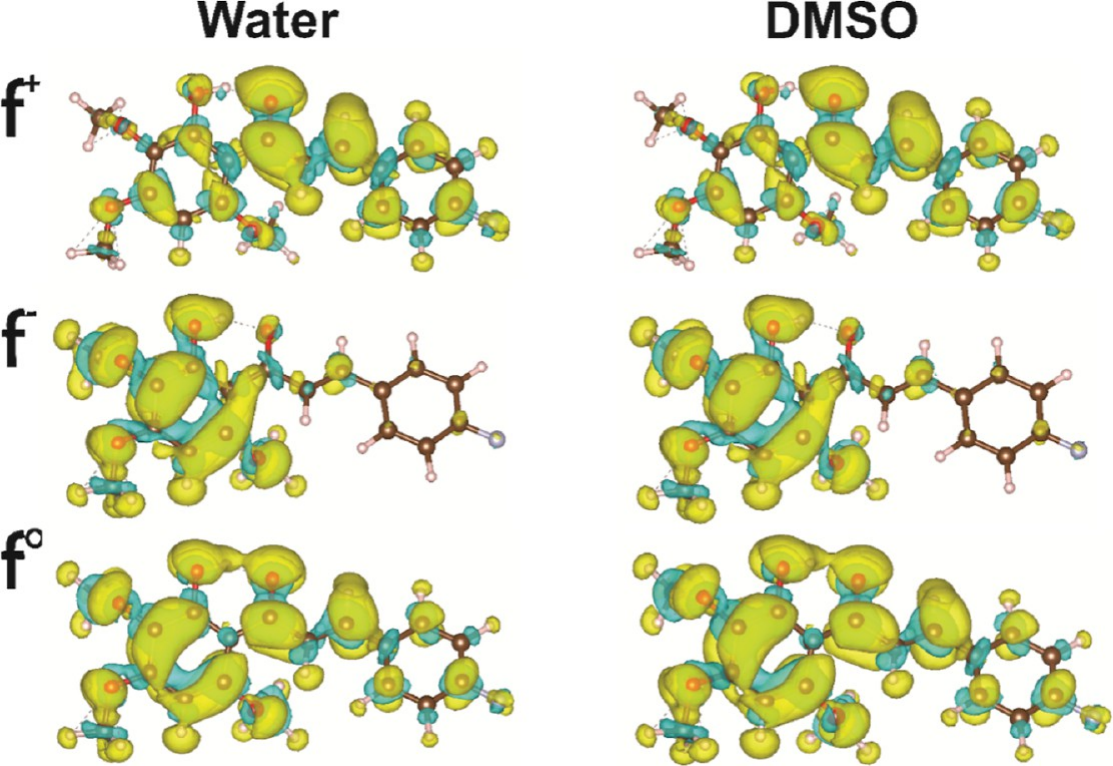
Electronic Fukui functions for the nucleophilic attack (*f* ^+^), electrophilic attack (*f* ^–^), and radical attack (*f* ^0^) for the chalcone CPN4F in (a) water and (b) DMSO environments.

Next, the condensed Fukui functions were computed using the Hirshfeld population analysis; the results for chalcone in water (CPN4F-W) are shown in Figure 13 and those for chalcone in DMSO (CPN4F-D) are shown in Figure 14: nucleophilic (*f*_*k*_^+^), electrophilic (*f*_*k*_^–^), and radical (*f*_*k*_^0^) attack. For both water and DMSO environments, the atoms that are most susceptible to nucleophilic attack are C25, O26, C27, C28, C31, C32, C33, C34, C36, and F41. By combining this result with that of the electronic function, it is evident that the B ring and the α,β-unsaturated structure are indeed the regions most prone to behave as electrophiles when interacting with a nucleophile. For the electrophilic attack, the atoms that are most susceptible are C1, C2, C3, C4, C5, C6, O8, O9, O10, O11, C13, C17, and C21 for both environments. This result aligns well with the electronic function, indicating that the regions most likely to donate electron density are ring A and the three methoxy groups. This observation is expected, as the HOMO is predominantly distributed over these atoms. When comparing radical attacks to nucleophilic or electrophilic attacks, the atoms of this compound are less likely to undergo such radical attack, preferring instead to act as electrophiles or nucleophiles. These same trends for susceptibility to nucleophilic and electrophilic attack were confirmed by the dual descriptor (Δ*f*) and the multiphilic index (Δω), the results of which are shown in Table 5. The atoms most prone to nucleophilic attack are C1, C2, C3, C4, C5, C6, O8, O9, O10, O11, C13, C17, and C21. The atoms most susceptible to electrophilic attack are C25, O26, C27, C28, C31, C32, C33, C34, C36, C38, and F41.

**Figure 13:**
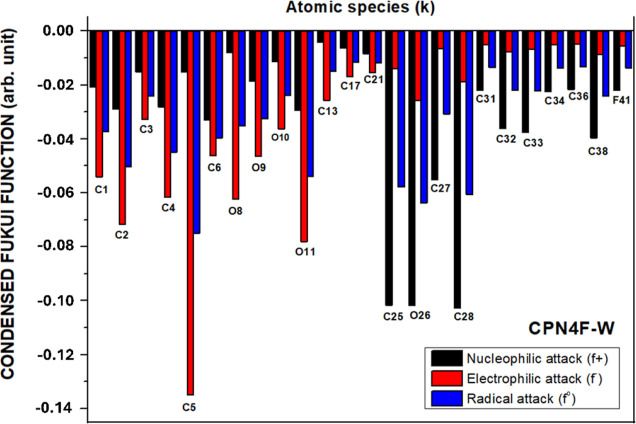
Condensed Fukui functions for the nucleophilic (*f*_*k*_^+^), electrophilic (*f*_*k*_^–^), and radical (*f*_*k*_^0^) attack for the chalcone in water (CPN4F-W).

**Figure 14:**
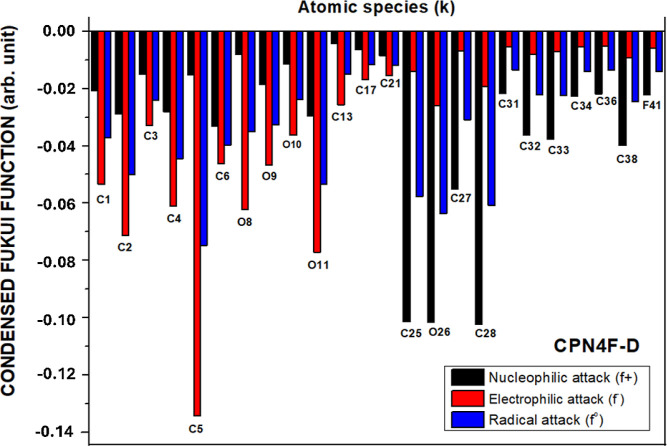
Condensed Fukui functions for the nucleophilic (*f*_*k*_^+^), electrophilic (*f*_*k*_^–^), and radical (*f*_*k*_^0^) attack for the chalcone in DMSO (CPN4F-D).

**Table 5:** Dual Descriptor (Δ*f*) and Multiphilic Index (Δω) Obtained for the Chalcone CPN4F in Water and DMSO Environments

	CPN4F-W		CPN4F-D	
atom	dual descriptor (Δ*f*)	multiphilic index (Δω)	dual descriptor (Δ*f*)	multiphilic index (Δω)
C1	0.033321	0.175246	0.032613	0.171424
C2	0.042761	0.224895	0.042579	0.223808
C3	0.017605	0.092591	0.01784	0.093772
C4	0.033562	0.176514	0.0329	0.172932
C5	0.11958	0.628912	0.118905	0.625
C6	0.013334	0.070128	0.013293	0.069872
O8	0.054455	0.286397	0.054089	0.284308
O9	0.027888	0.146672	0.028046	0.147418
O10	0.024927	0.1311	0.024936	0.131071
O11	0.048689	0.256072	0.047713	0.250794
C13	0.021553	0.113355	0.021424	0.112611
C17	0.010489	0.055165	0.010501	0.055196
C21	0.007084	0.037257	0.007057	0.037094
C25	–0.08763	–0.46089	–0.08746	–0.45973
O26	–0.07584	–0.39889	–0.07569	–0.39782
C27	–0.04854	–0.25528	–0.04812	–0.25291
C28	–0.08378	–0.44062	–0.08318	–0.43721
C31	–0.01674	–0.08805	–0.01641	–0.08626
C32	–0.02837	–0.14922	–0.02802	–0.1473
C33	–0.0307	–0.16144	–0.03046	–0.16008
C34	–0.01738	–0.09141	–0.01719	–0.09037
C36	–0.01673	–0.088	–0.01657	–0.08711
C38	–0.03093	–0.16266	–0.03062	–0.16093
F41	–0.01644	–0.08646	–0.01626	–0.08544

Finally, the Molecular Electrostatic Potential (MEP) for both water and DMSO environments computed at the B3LYP/6-311++G­(d,p) level of theory is shown in Figure 15. As shown in Figure 15, the color-coded regions indicate various charge distributions: the red regions represent a negative charge distribution, the yellow regions indicate a partial negative charge distribution, the green regions denote a neutral charge distribution, the light-blue regions indicate a partial positive charge, and the blue regions represent a positive charge distribution. It is evident that changing the solvent type (protic or aprotic) did not affect the charge distribution of the CPN4F chalcone. Notably, the oxygen atoms in the methoxyl, hydroxyl, and carbonyl groups show a negative charge distribution (red regions). This finding aligns with previous observations, as ring A exhibits a higher concentration of negative charge available for donation. Additionally, a red-to-yellow distribution can be seen on rings A and B of the chalcone. This is related to both the π-electron density and the delocalization of electron density from the methoxyl groups (ring A), as well as the α,β-unsaturated structure (ring B). These features suggest the potential for π interactions with other molecules, as discussed previously.

**Figure 15:**
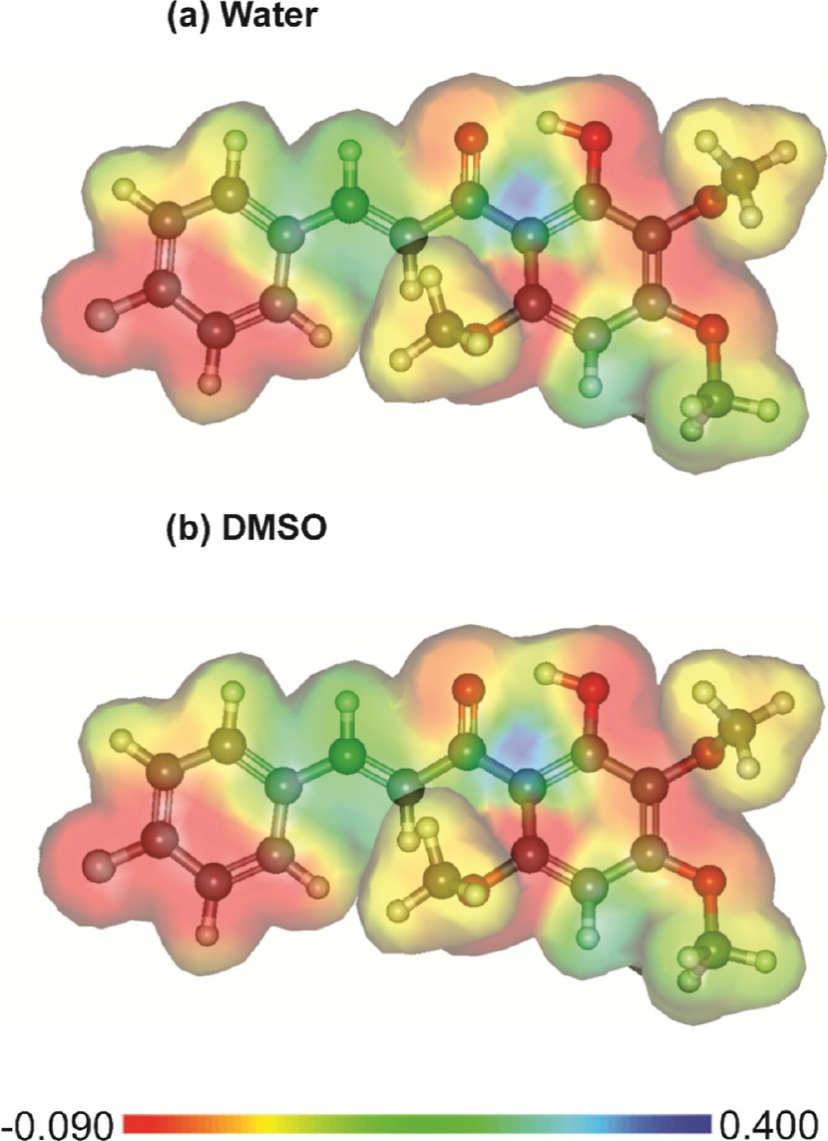
Molecular electrostatic potential (MEP) calculated at the B3LYP/6-311++G­(d,p) computational level for the chalcone CPN4F in (a) water and (b) DMSO.

### 3.2 Cytotoxicity in Host LLC-MK2 Cells

To assess toxicity in host cells, as well as to determine the compound’s selectivity for *T. cruzi*, the MTT reduction assay was carried out. The chalcone CPN4F showed toxicity at all the concentrations tested, except 15.6 μM, with percentages of viable cells ranging from 20.38 to 76.55% (Table 6 and Figure 16). In addition, the CC_50_ value was estimated at 99.26 μM for the chalcone (Table 6), and 502.60 μM for the benznidazole.

**Table 6:** Viability Percentages of Host Cells Treated with CPN4F^a^

concentration (μM)	CPN4F
control	99.68 ± 0.44
vehicle	94.80 ± 0.89
1000	20.38 ± 2.05*
500	25.41 ± 1.31*
250	38.14 ± 2.12*
125	46.75 ± 4.38*
62.5	57.50 ± 3.86*
31.2	76.55 ± 2.08*
15.6	98.12 ± 1.12

a Data are presented as mean ± SEM. Statistical analysis was performed by one-way ANOVA with Dunnett’s post-test. *p < 0.05 in relation to the control group.

**Figure 16:**
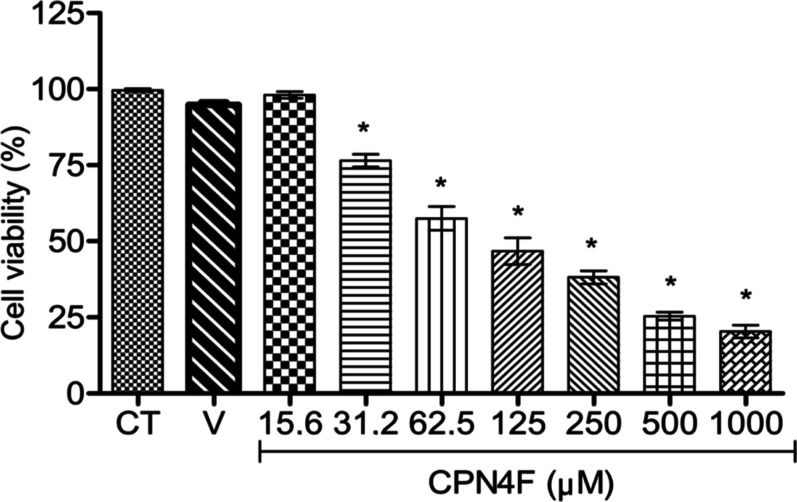
Cytotoxicity of CPN4F in LLC-MK2 cells. Data are presented as mean ± SEM. Statistical analysis was performed by one-way ANOVA with Dunnett’s post-test. *p < 0.05 in relation to the control group.

The potential trypanocidal and cytotoxic effects of CPN4F may be attributed to its α,β-unsaturated system, which confers high reactivity to this group of molecules.^80^ Furthermore, substitutions on the A and B rings of chalcones have been shown to either increase or decrease the biological activity of this group of compounds.^81^

A study by Lunardi et al.^82^ showed that the addition of highly reactive halogenated groups, such as chlorine and fluorine, to the A and B rings of chalcones intensified the biological effect of these molecules, which is very little described for models of antiparasitic activity, especially in *T. cruzi*.

### 3.3 Effect on Epimastigote Forms of *T. cruzi*

The antiproliferative effect of CPN4F on the epimastigote forms of *T. cruzi* was assessed by determining the percentage of viable cells (Table 7). At 24 h (Figure 17A), the percentage of viable epimastigote forms ranged from 21.40% to 72.83%. This effect was accentuated at 48 h (Figure 17B), with the percentage of viable epimastigotes ranging from 17.09% to 76.50%, and at 72 h, with the percentage of viable parasites ranging from 14.69% to 60.29% (Figure 17C). The IC_50_ values were used to determine the inhibition of parasite multiplication. At 24, 48, and 72 h, the values were 356.70 μM, 149.30 μM, and 22.95 μM, respectively. For benznidazole, the values were 115.10 μM, 37.24 μM, and 22.08 μM at 24, 48, and 72 h, respectively (Table 7).

**Table 7:** Cv Percentage of Epimastigote Forms Treated with CPN4F^a^

CPN4F (μM)	24 h	48 h	72 h
control	100.00 ± 2.48	100.00 ± 2.03	100.00 ± 2.24
vehicle	98.61 ± 4.92	94.87 ± 1.05	102.80 ± 3.16
1000	21.40 ± 5.28*	17.09 ± 1.18*	14.69 ± 4.45*
500	36.95 ± 8.11*	35.09 ± 3.80*	30.66 ± 8.25*
250	50.48 ± 4.83*	48.91 ± 2.55*	33.93 ± 7.63*
125	57.07 ± 4.30*	63.84 ± 5.74*	41.08 ± 8.94*
62.5	61.70 ± 4.60*	72.04 ± 4.71*	47.58 ± 8.10*
31.2	72.83 ± 4.36*	76.50 ± 4.58*	53.44 ± 6.17*
15.6	85.80 ± 2.50*	89.74 ± 4.90	60.29 ± 7.48*

a Data are presented as mean ± SEM. Statistical analysis was carried out using one-way ANOVA with Dunnett’s post-test. *p < 0.05 in relation to the control group.

**Figure 17:**
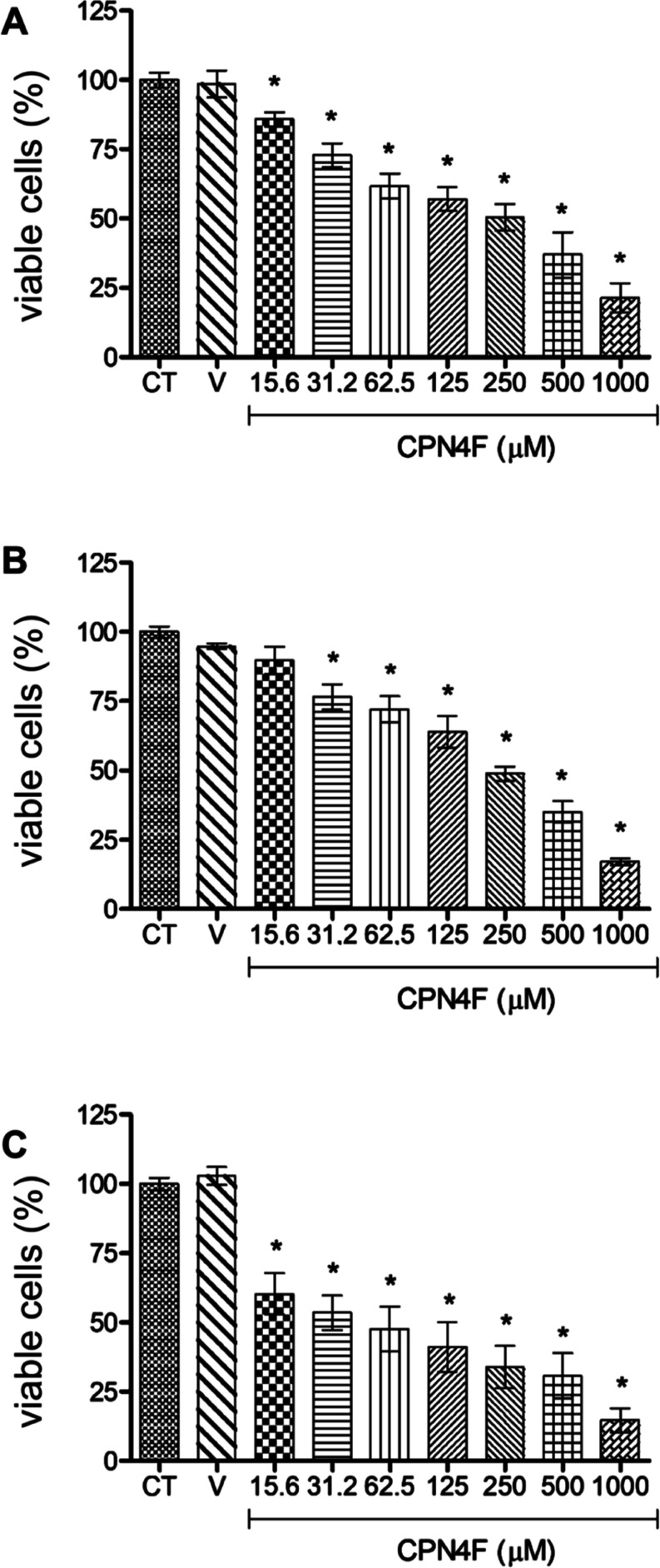
Antiproliferative effect of CPN4F on epimastigote forms of *T. cruzi* at (A) 24 h, (B) 48 h, and (C) 72 h. Data are presented as mean ± SEM. Statistical analysis was performed by one-way ANOVA with Dunnett’s post-test. *p < 0.05 in relation to the control group.

The chalcone CPN4F had an effect comparable to that of BZN on the epimastigote forms. This is an important discovery, as these forms of multiplication, although not present in the vertebrate host, are used as screening in experimental models of CD. This reinforces the biological potential of chalcones.^38^

### 3.4 Effect on Trypomastigote Forms of *T. cruzi*

For chalcone CPN4F (Figure 18), the percentage of live trypomastigote forms was 61.22% at the lowest concentration and 0.00% at the four highest concentrations (Table 4), with an estimated LC_50_ of 32.54 μM (Table 4). For BZN, the LC_50_ was estimated at 161.40 μM (Table 8).

**Figure 18:**
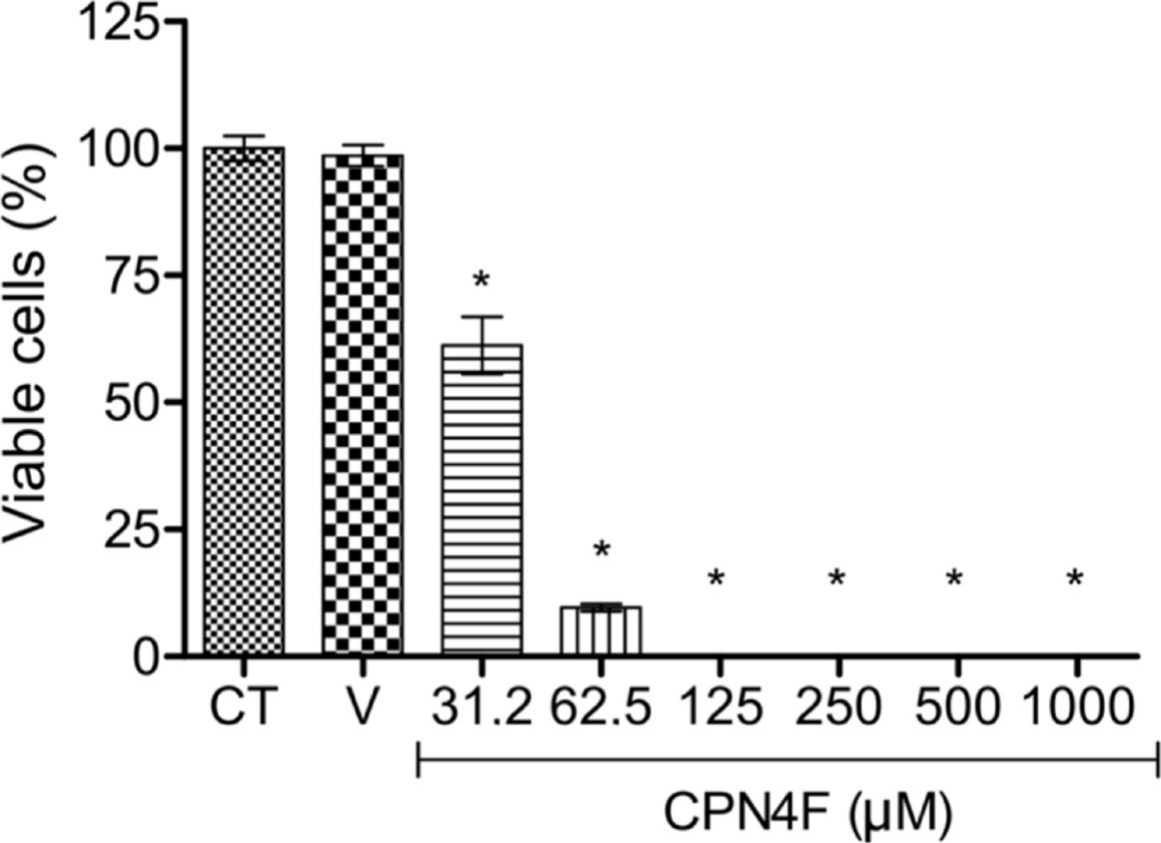
Trypanocidal effect of CPN2F on *T. cruzi*. Data are presented as mean ± SEM. Statistical analysis was performed by one-way ANOVA with Dunnett’s post-test. *p < 0.05 in relation to the control group.

**Table 8:** Cv Percentage of Trypomastigote Forms Treated with CPN4F^a^

concentration (μM)	CPN4F
control	100.00 ± 2.45
vehicle	98.61 ± 2.10
1000	0.00 ± 0.00*
500	0.00 ± 0.00*
250	0.00 ± 0.00*
125	0.00 ± 0.00*
62.5	9.62 ± 0.71*
31.2	61.22 ± 5.61*

a Data are presented as mean ± SEM. Statistical analysis was performed by one-way ANOVA with Dunnett’s post-test. *p < 0.05 in relation to the control group.

With the results of cytotoxicity in host and trypomastigote cells, it was possible to determine the selectivity of the substances. The SI values were 3.05 and 3.11 for CPN4F and benznidazole, respectively (Table 9). In trypomastigote forms, CPN4F was significantly more effective than BZN. This finding is crucial as trypomastigote forms play a major role in the acute phase of CD as well as in the infection of new hosts and the spread of the disease.^38^

**Table 9:** Effect on Host Cells, Epimastigotes, and Trypomastigotes of *T. cruzi* ^a^

	24 h	48 h	72 h
CPN4F			
CC_50_	99.26 ± 14.98	-	-
IC_50_	356.70 ± 88.20	149.30 ± 29.20	22.95 ± 8.70
LC_50_	32.54 ± 1.19	-	-
IS	3.05	-	-
BZN			
CC_50_	502.60 ± 57.80	-	-
IC_50_	115.10 ± 16.32	37.24 ± 5.55	22.08 ± 2.92
LC_50_	161.40 ± 31.80	-	-
IS	3.11	-	-

a CC_50_: concentration capable of reducing the viability of host cells by 50%; IC_50_: concentration capable of inhibiting 50% of the proliferation of epimastigote forms; LC_50_: concentration capable of killing 50% of trypomastigote forms.

The World Health Organization (WHO) states that molecules with a selectivity index (SI) greater than 50 are promising for treating CD.^83^ Despite CPN4F having a superior effect compared with benznidazole, its selectivity is still low due to its considerable toxicity to host cells.

The potential trypanocidal effect of chalcones may be attributed to their interaction with the enzyme cruzain. Cruzain performs functions that are related to the infectivity, differentiation, and multiplication of the parasite.^84^ Previous studies have linked the in vitro effect of quinolines, aziridines, chalcones, and hydrazones on strains of *Leishmania* sp. and *Trypanosoma* sp. to the inhibition of cruzain enzyme activity in these organisms.^85–87^

In summary, the CPN4F compound demonstrated a CC_50_ of 99.26 (±14.98) μM in LLC-MK2 cells, while the calculated IC_50_ values for epimastigotes ranged from 356.7 ± 88.2 μM (in 24 h), 149.3 ± 29.2 μM (in 48 h), and 22.95 ± 8.7 μM (in 72 h), yielding an SI of 3.05 after 24 h (Table 9). This result was analogous to that observed for BZN (3.11). These results suggest that, while CPN4F demonstrates antiproliferative activity against *T. cruzi*, its selectivity against host cells is moderate. Consequently, further experimentation in diverse mammalian cell lines and in vivo models is imperative to substantiate the selective safety observed in vitro.

### 3.5 Molecular Docking Analysis

Molecular recognition is crucial for comprehending ligand–receptor interactions and validating molecular docking simulations. It enables an optimized analysis of hydrogen bond donor and acceptor groups involved in these interactions.^88^ Recently published studies have investigated inhibiting the proliferation of *T. cruzi* using in silico techniques. Molecular recognition has been widely applied in analyses of ligand–receptor interactions with targets in the parasite’s evolutionary cycle. These studies have shown excellent correlation with in vitro tests.^89,90^

The selection of the optimal route for inhibiting parasite proliferation is based on a systematic alignment between affinity energy (Δ*G* < −6.0 kcal mol^–1^) and intermolecular interaction strengths. The rmsd range should express high specificity for the target protein (rmsd < 2.0 Å), and the intermolecular interaction strengths should be between strong (distance between 2.5 and 3.1 Å) and weak (distance >3.1 Å).^63–65,91^

The simulations were parametrized by the redocking protocol, which involves the ligands cocrystallized with the targets. These simulations can be viewed in Figure 19. For the cruzain enzyme, it was possible to observe that the best pose of the redocking showed an rmsd of 0.743 Å (Figure 19A). The greatest displacement was located in the aliphatic side chain of the nitro-substituted base (green color). The consistent parametrization of the cruzain docking protocol is of great importance, as the protein is directly related to the degradation of proteins essential for the parasite’s survival.^24^ With regard to TcGAPDH, its redocking was executed by employing the cocrystallized inhibitor BRZ, resulting in an rmsd deviation of 1.140 Å. The redocking complex (green color) was positioned in relation to the reference pose (gray color), which corresponded to the same binding domain. This reference pose was linked to an enzyme that plays a crucial role in NAD^+^-dependent oxidative phosphorylation of metabolic products, thereby facilitating the proliferation of the parasite within host cells.^23^ Conversely, the binding of the QUM inhibitor to the binding site of trypanothione reductase exhibited low specificity in redocking, as evidenced by high rmsd deviations. This finding suggests that the compound demonstrates low selectivity for new inhibitor candidates within this pathway and low reliability in the reproducibility of the molecular docking simulation steps.^64^

**Figure 19:**
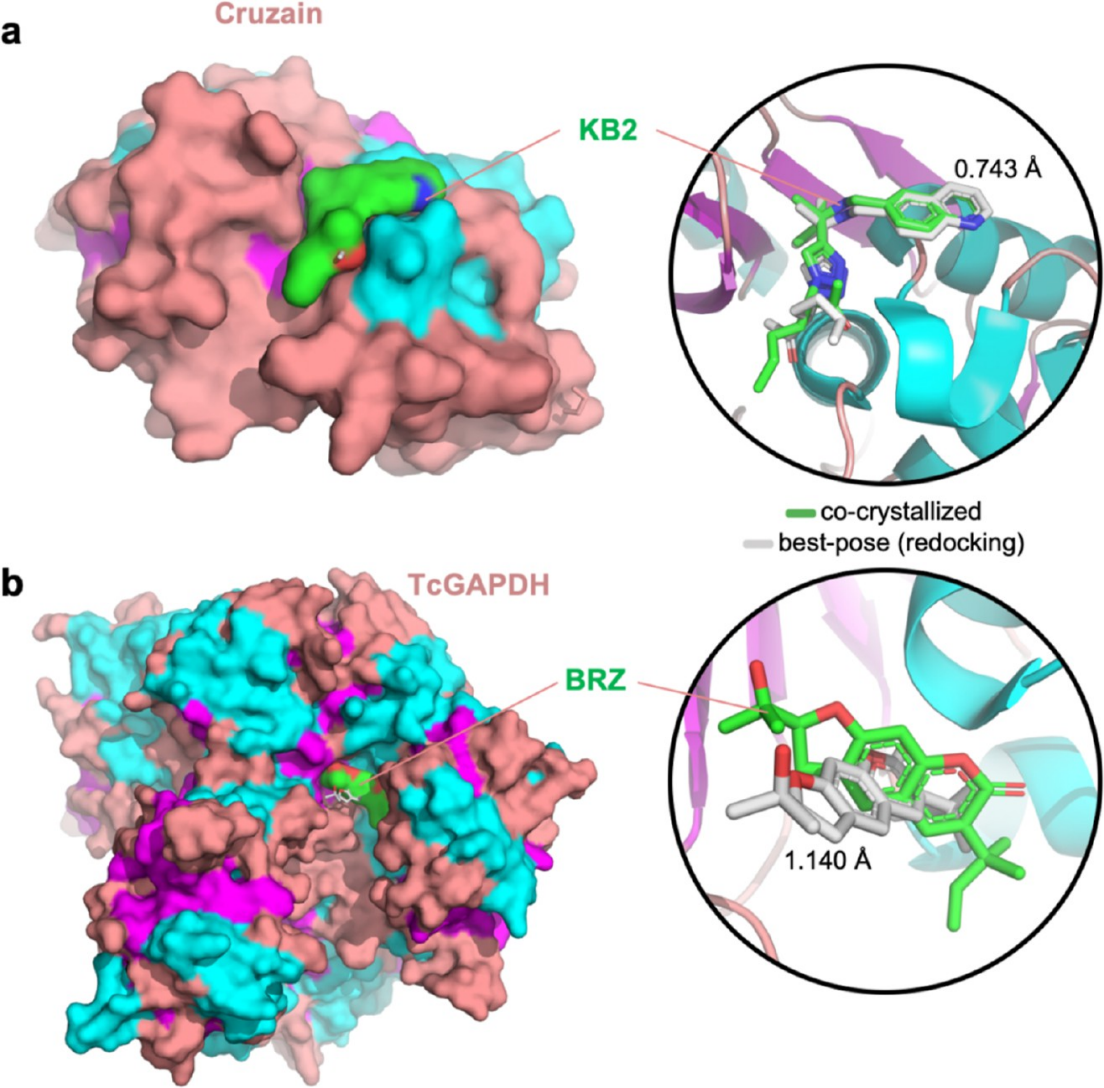
(A) Three-dimensional visualization of the cruzain protein with the KB2 inhibitor complexed and enlarged image showing the best pose of redocking (gray) in relation to the cocrystallized inhibitor (green). (B) Three-dimensional visualization of the TcGAPDH protein with the complexed BRZ inhibitor and enlarged image showing the best pose of redocking (gray) in relation to the cocrystallized inhibitor (green).

According to the findings of this preliminary investigation, molecular recognition appears to be a viable approach for identifying novel cruzain and TcGAPDH inhibitors, exhibiting a reasonable degree of reliability.^64,92,93^ Given its aromatic nature, it is evident that chalcone can engage in a series of intermolecular interactions, akin to those of other aromatic or heteroaromatic inhibitors of these enzymes, such as BZN.^21,90^

When analyzing the affinity energies expressed in the graph in Figure 20, it is evident that the reference drug BZN has the best free energy order (Δ*G* = −7.6 kcal mol^–1^) for the trypanothione reductase target compared to the chalcone CPN4F (Δ*G* = −6.0 kcal mol^–1^) and the cocrystallized inhibitor (QUM). When analyzing the affinity energies expressed in the graph in Figure 20, it is evident that the reference drug BZN has the best free energy order (Δ*G* = −7.6 kcal mol^–1^) for the trypanothione reductase target compared to the chalcone CPN4F (Δ*G* = −6.0 kcal mol^–1^) and the cocrystallized inhibitor (QUM). However, all values are still within the ideal free energy limit for effective binding to a receptor (Δ*G* < −6.0 kcal mol^–1^).^43^ However, the chalcone exhibited a higher affinity energy (Δ*G* = −6.3 kcal mol^–1^) than BZN (Δ*G* = −6.0 kcal mol^–1^) and the inhibitor (KB2) against the cruzain target. In regards to the TcGPDH target, the ligand inhibitor BZR exhibited a stronger affinity to the receptor (Δ*G* = −8.0 kcal mol^–1^) compared to CPN4F. However, CPN4F demonstrated a higher free energy order than the BZN drug, with a calculated Δ*G* value of −7.0 kcal mol^–1^, and was more effective in coupling to other targets (Figure 20).

**Figure 20:**
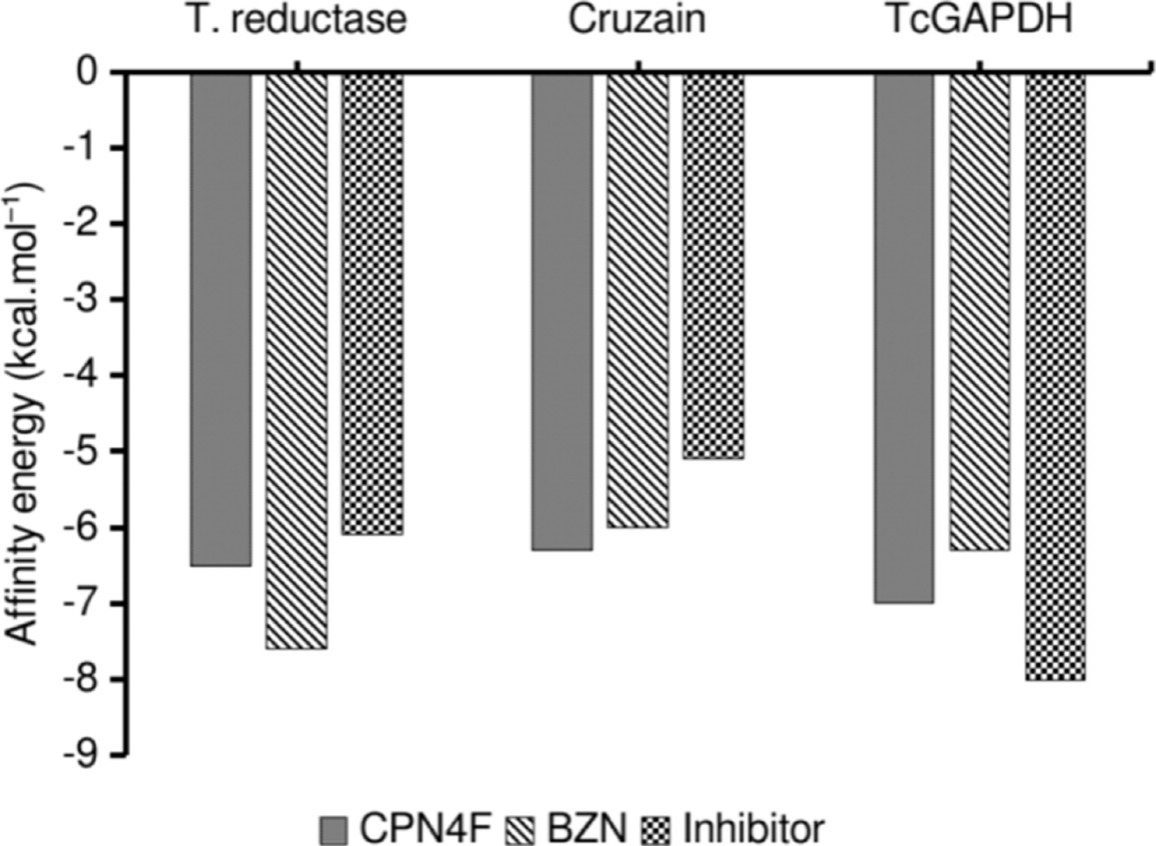
Affinity energy values calculated during the molecular docking simulations of the CPN4F ligands, the BZN drug, and the inhibitors cocrystallized in their appropriate targets: QUM-T.reductase, KB2-cruzain, and BZR-TcGAPDH.

According to the findings of previous studies, oxidative stress induced by chalcones appears to be directly linked to cell membrane degradation, which bears a resemblance to the process of necrosis. However, it should be noted that this process is more thoroughly characterized and observed in the context of cancer cells compared to cells infected by trypanosomatids.^29,94^ These processes result in damage to the host cell’s DNA and impairment of the mitochondrial matrix.^95–98^ The present study is consistent with other investigations that have demonstrated the occurrence of necrosis, oxidative stress, and cell death events induced by chalcones, thereby reinforcing the trypanocidal potential of these molecules.^89^

The docking simulations against the cruzain target demonstrated that the ligands exhibited common interactions despite occupying different sites on the protein (Figure 21). The analysis indicates that the CPN4F chalcone and the BZN ligand (Figure 21B) exhibit interactions that are analogous to those observed with the Gln 19A residue, which is also analogous to the cocrystallized KB2 ligand (Figure 21A). This result suggests that chalcone may act as a cruzain inhibitor, similar to previously characterized inhibitors of the enzyme, such as KB2 and BZR, by binding to the same binding domain and competing for the same inhibitory effect.^24^

**Figure 21:**
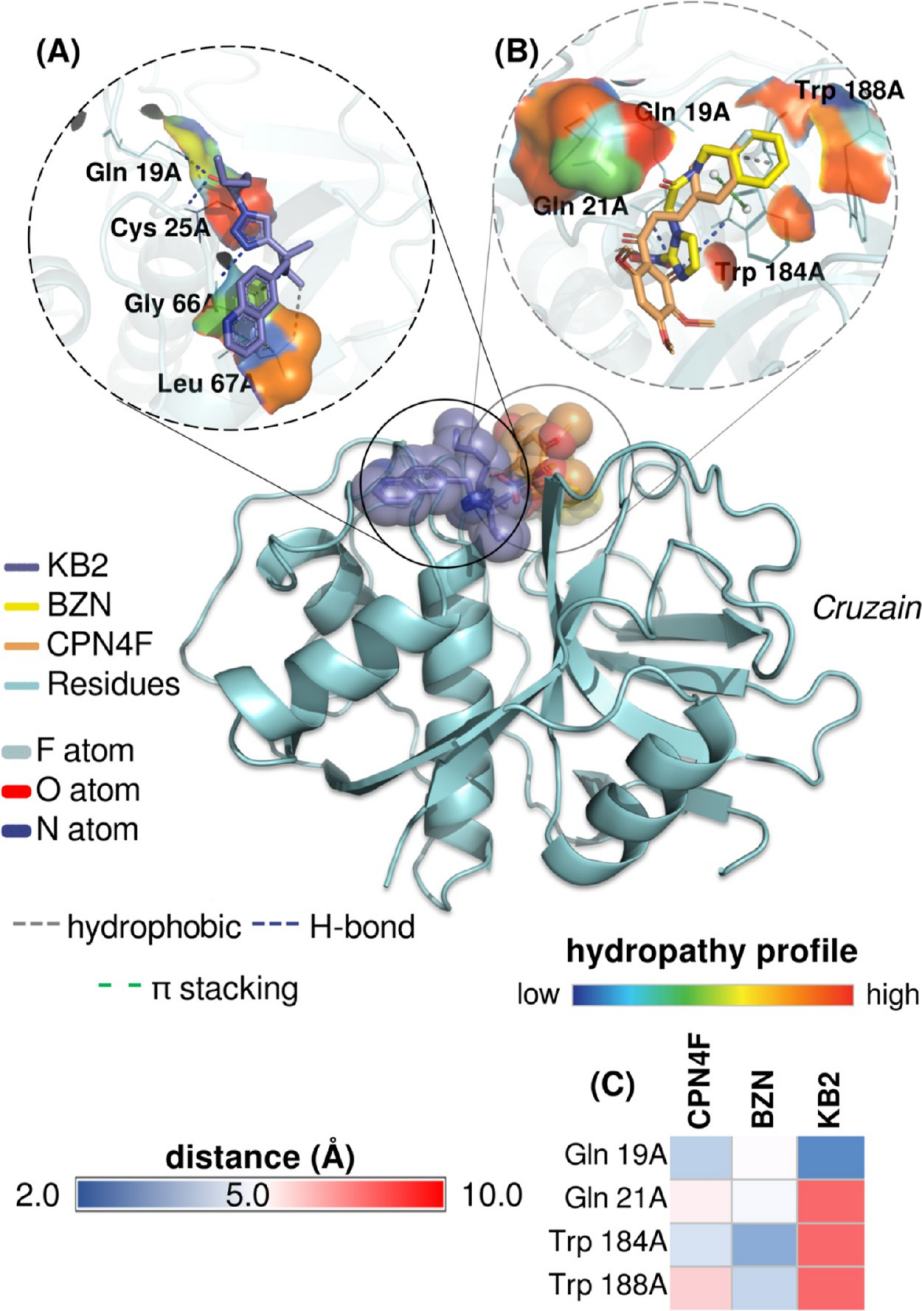
Three-dimensional visualization of the docking of the (A) inhibitor to KB2 (purple), (B) the drug BZN (yellow), and the chalcone CPN4F (orange) on the cruzain target; (C) heatmap of the interaction distance–force relationship.

This interaction occurs in a region with a high hydropathic index (yellow–red spectrum). In Figure 11C, it can be observed that the KB2 ligand forms a stronger H-bond (distance = 2.28 Å) compared to the CPN4F and BZN ligands. The distances calculated for CPN4F and BZN are 3.41 Å and 4.22 Å, respectively (Table S1). Additionally, the ligands interact with the aromatic amine groups of the Trp 184A residue, with calculated distances of 3.15 Å and 2.83 Å (respectively, CPN4F and BZN), highlighting the π-stacking interaction that the ligand has with the residue (Figure 21B). CPN4F can modulate the activity of the cruzain target by interacting with the KB2 ligand against the Gln 19A residue of the active site.

After the independent simulations carried out for the trypanothione reductase target, it was possible to observe that the chalcone CPN4F and the reference drug BZN bound to different sites from each other and from the cocrystallized ligand QUM. In the binding site of the QUM inhibitor, hydrophobic interactions predominate (interactions using the π electronic density for the donor or acceptor with HOMO and LUMO, respectively), where the surface of the amino acid residues has a low hydropathic index (blue tint spectrum), with a strong contribution from the aromatic side chain of the Trp 22 residue (Figure 22A), where the aromatic substructure of the ligand interacted with the residue over a minimum distance of 3.47 Å (Table S1), a range characteristic of weak interactions (distance between 3.1 and 3.55 Å).^91^ The interaction surfaces of the CPN4F and BZN ligands have a high hydropathic index when compared to the inhibition site (yellow–red spectrum). Here, it was possible to observe that the reference drug BZN made a weak hydrogen bond interaction with the Arg 288A residue (Figure 22B) with a calculated distance of 3.27 Å (Table S1), while the chalcone CPN4F showed a strong hydrogen bond interaction with the His 73 residue (Figure 22C) with an estimated distance of 1.92 Å (Table S1).

**Figure 22:**
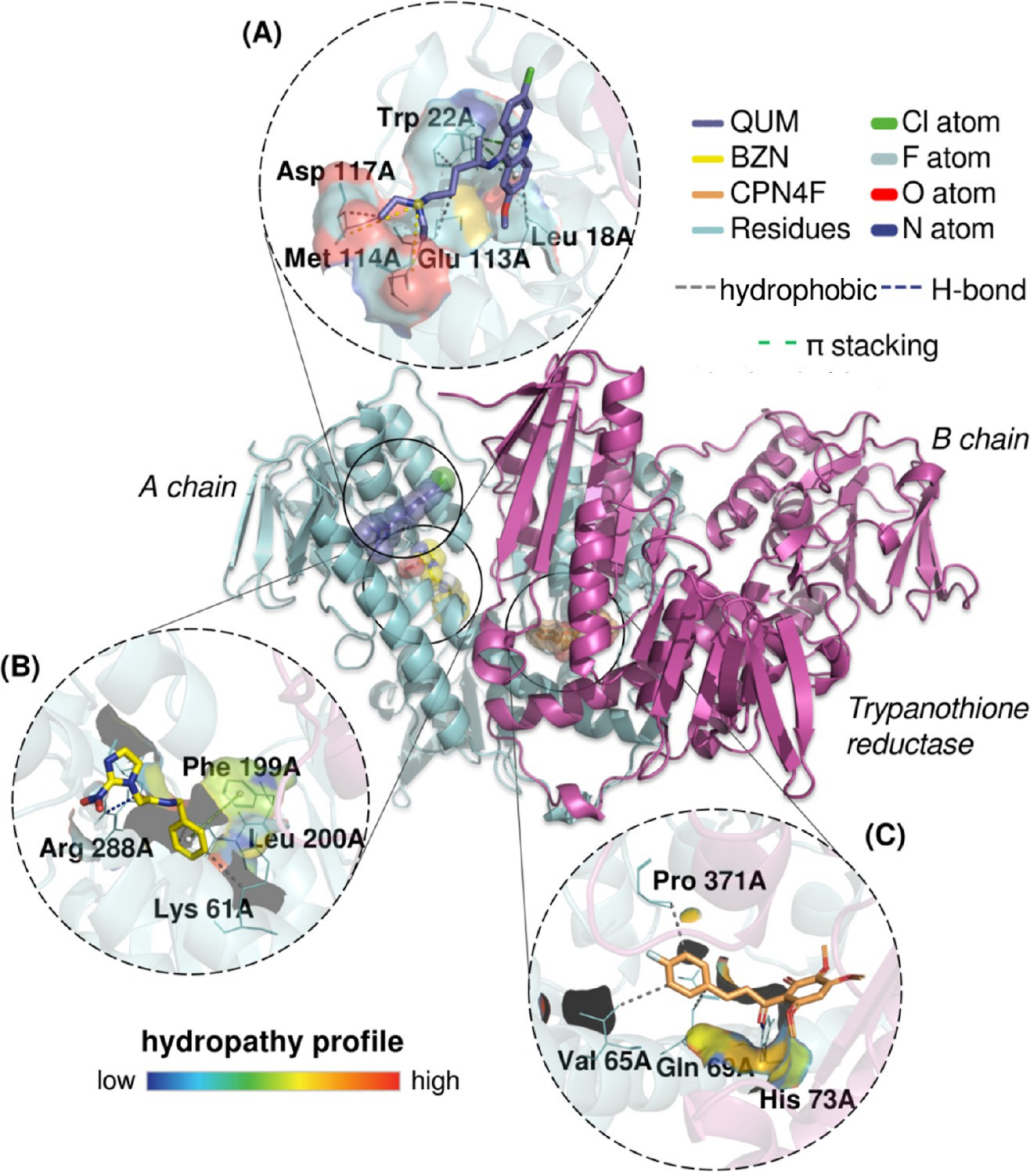
Three-dimensional visualization of the coupling of the inhibitor (A) QUM (purple), (B) the drug BZN (yellow), and (C) the chalcone CPN4F (orange) to the A chain of trypanothione reductase and its hydropathy profile.

When the docking simulations against the TcGAPDH target were evaluated, it was possible to observe that both the CPN4F ligand and the BZN control ligand interact with the C-chain active site occupied by the BZR inhibitor (Figure 23A). It is interesting to note the similarity of the fit between the CPN4F ligand and the BZR inhibitor, mainly due to the calculated distance to the Cys 166C and Thr 167C residues between 2.5 and 3.0 Å (Figure 23B), a distance range that characterizes strong H-bond interactions,^91^ regions of moderate hydropathic surface (green color spectra). On the other hand, BZN interacts more strongly with residues Ile 731C, His 912C, Ser 965C, and Glu 1054C than with the most frequent residues among the CPN4F and BZR ligands, with calculated distances between 3.0 and 4.16 Å (Figure 23B).

**Figure 23:**
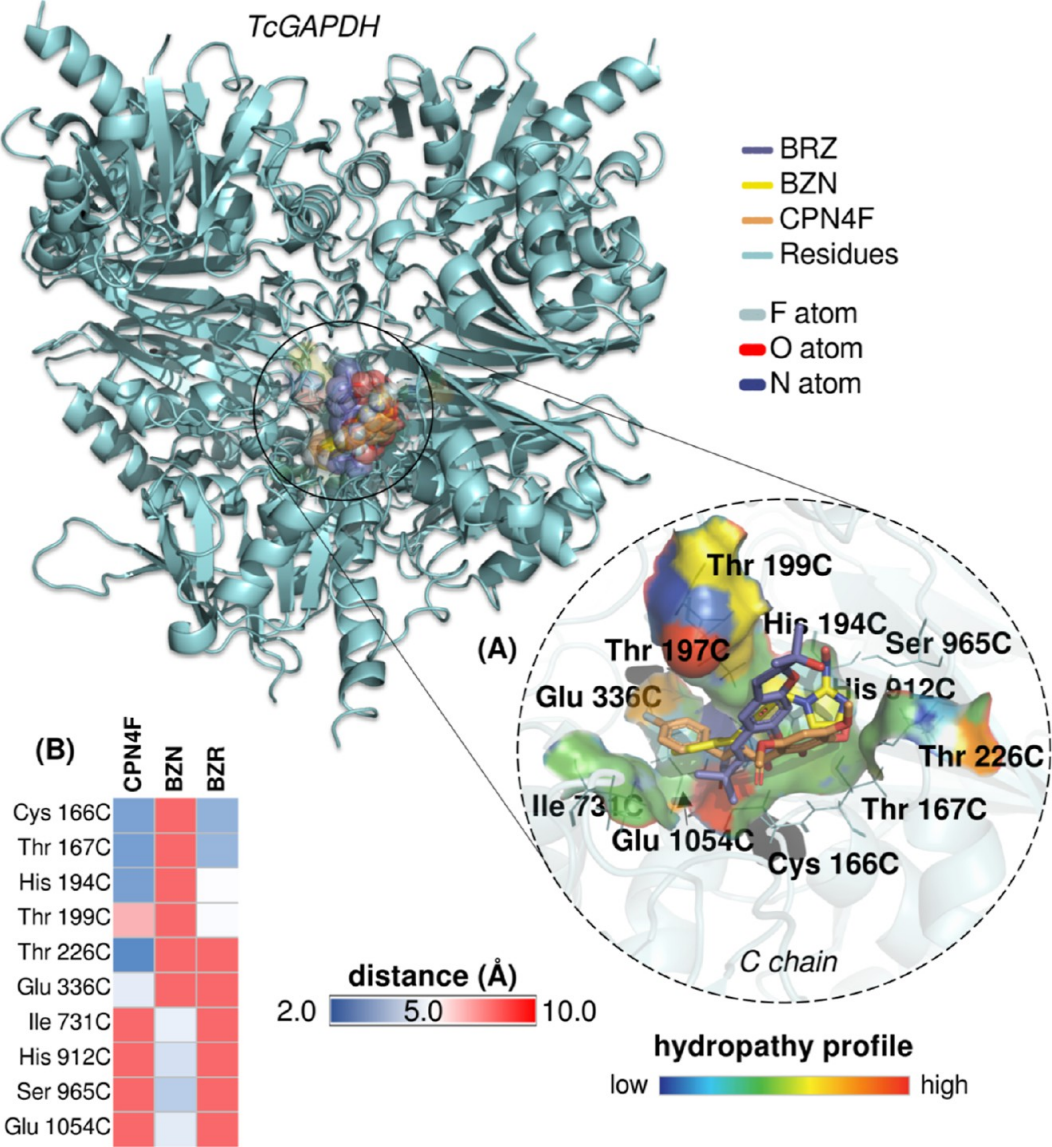
(A) Three-dimensional visualization of the coupling of the inhibitor BZR (purple), the drug BZN (yellow), and the chalcone CPN4F (orange) to the TcGAPDH target and (B) heatmap of the interaction distance–force relationship.

It was possible to observe that the chalcone CPN4F showed the cruzain target as a favorable route against *T. cruzi* proliferation, due to the free energy order being higher than the control ligands BZN and KB2, within the statistical parameters that infer good specificity for the selected targets (rmsd < 2.0 Å). The molecular docking simulations showed that the common interactions that the ligand has with BZN reveal a condition of similar efficacy to the drug.

### 3.6 Molecular Dynamics Simulation

The objective of MD in this study is to evaluate the stability of the formed complex, as evidenced by the persistence of the ligand in the region where it docked in molecular docking calculations. This validation process serves to complement the study.^99^ rmsd analysis is a principal tool for the evaluation of key aspects of MD simulations, including the stability of protein–ligand complexes, dynamically flexible complexes, proteins with multiple conformations, and conformational transitions. Previous studies have reported that using the leapfrog integrator and the Parrinello–Rahman method as a barostat results in more stable simulations.^100,101^ These methods were employed in this study, contributing to the observed good results. The use of the CGenFF server to obtain the parameters of the CPN4F molecule was also a good choice due to the high quality of the parameters generated.^102^

Regarding the rmsd results for the CPN4F-CRUZAIN complex, similar conformational changes were observed in the three calculated MD runs, which, at 275 ns, showed conformational stability with an average variation in the order of 2 Å (0.20 nm), remaining stable with low fluctuation until the end of the simulation, indicating stability in the complex. As demonstrated in Figure 24, an examination of the root-mean-square deviation (rmsd) values reveals a variation of approximately 1.25 Å (0.125 nm) in the initial frame; this is due to thermal equilibration. Subsequent to this, the system does not present a conformational change until the 110 ns mark, which shows a variation from 1.5 Å (0.15 nm) to 2 Å (0.2 nm). Thereafter, the system remains in this conformation until 200 ns, where it again presents a conformational increase varying from 2 Å (0.2 nm) to 2.3 Å (0.23). Thereafter, the system stabilizes at 275 ns, remaining in this conformation until the end of the MD. During the MD, the complex exhibited a variation of 1.2 Å (0.12 nm), indicative of a semirigid protein structure with minor variations shortly after equilibrium over 500 ns observed in the three runs. The findings underscore the significance of the calculation time, as the most stable conformational structure of the complex was observed at 275 ns. The ligand maintained its position within the coupling region of molecular docking, thereby demonstrating notable stability in this domain.

**Figure 24:**
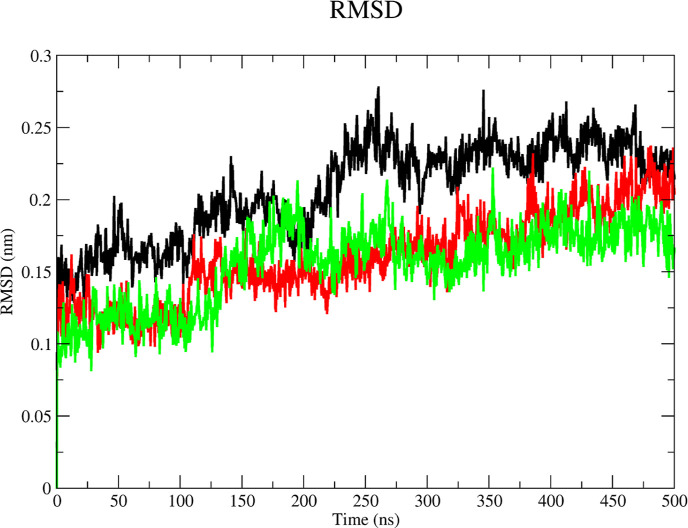
Determination of the root-mean-square deviation (rmsd) for the CPA4F-CRUZAIN complex at 500 ns in triplicate in black, red, and green colors for Run 1, Run 2, and Run 3, respectively.

Figure 25 illustrates the RMSF of the complex CPN4F-CRUZAIN, which is represented in red. It has been established that the terminal residue and surface loop regions exhibit greater flexibility, while the protein core demonstrates a more constrained mobility.^103^ It is possible to observe fluctuations in the order of 1.9 Å (0.19 nm) in the N-terminal residue (Ala 1), in the loop residue Asp 18 fluctuations in the order of 2.1 Å (0.21 nm), in the loop residue and beginning of α helix Asp 60 2.2 Å (0.22 nm), and the most pronounced fluctuations were in the loop residues Gly 96 and Thr 103 with fluctuations in the order of 3.0 Å (0.30 nm) and 2.9 Å (0.29 nm), respectively. This finding indicates that these amino acid residues exhibit dynamic flexibility, thereby providing a rationale for the previously observed rmsd variation. However, it is noteworthy that these amino acid groups, with the exception of Gln 19, do not demonstrate direct interactions with the studied ligand.

**Figure 25:**
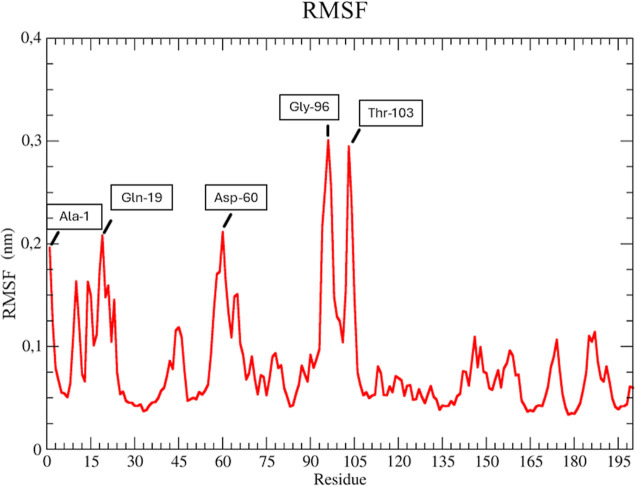
Determination of root mean square fluctuation (RMSF) for the CP4NF-CRUZAIN complex.

The results of the energy components for Δ*G*_bind_ in relation to the CP4NF-CRUZAIN system indicate significant results in the ligand interactions with the target protein. The *E*_vdW_ term yielded a value of −24.85 kcal/mol, while the energy *E*_ele_ presented a value in the order of −1.69 kcal/mol. This finding indicates a modest increase in electrostatic interactions within the system. With regard to the polar solvation energy (*G*_GB_), the contribution was in the order of 13.75 kcal/mol, while the nonpolar solvation energy (*G*_SA_) was −3.05 kcal/mol, as can be seen in Table 11. In relation to the system entropy term (−*T*Δ*S*), a value of 4.98 kcal/mol was observed. In analyzing the contributions of the various terms, it is noteworthy that the term associated with the *E*_vdW_, which exerted the most significant favorable influence on the calculations, was accompanied by a slight favorable contribution from the terms *E*_ele_ and *G*_SA_. Conversely, the polar contributions (*G*_GB_) proved to be disadvantageous, exerting a substantial influence on the calculation of the Δ*G*_bind_ value. This finding suggests that the complex exhibits favorable interactions and, consequently, good stability, as indicated by a binding affinity of −10.86 ± 3.89 kcal/mol.

Hydrogen bonds play a fundamental role in proteins, directly linked to the stability of β-sheets and α-helices.^104,105^ In this regard, it is essential to present information on the hydrogen bonds formed between amino acid residues and ligands, which may elucidate the responses for the stabilization of the complex formed between CPN4F and cruzain. The occupancy percentage indicates the frequency with which the bond occurs during the molecular dynamics simulation, in relation to the time of 500 ns.

Amino acid residues are identified by the protein chain, their three-letter code, and residue number. As illustrated in Figure 26, the figure displays only bonds with occupancy levels greater than 5%, exhibiting a total of 16 distinct interactions. The analysis indicates that the most prevalent hydrogen bond is observed between the ligand and chain A residue Trp 184, with an occupancy of 41.96%. This observation indicates that this bond plays a crucial role in maintaining the stability of the complex formed.^105^ A variety of hydrogen bonds have been observed between ligands and high-occupancy residues, including A:Trp 188 (34.53%), A:Gln 19 (29.62%), A:Gly 20 (24.97%), and A:Gly 23 (23.63%). The remaining residues, as depicted in Figure 26, with occupancies below 20% are A:Gly 21, A:Met 145, A:His 162, A:Gln 19, A:Cys 25, A:Cys 22, and A:Ser 183, functioning as acceptors. In contrast, residues A:Gly 20, A:Trp 184, A:Met 145, and A:His 162 act as donors. These bonds indicate that these amino acid residues are involved in important interactions in the protein–ligand system.^104^

**Figure 26:**
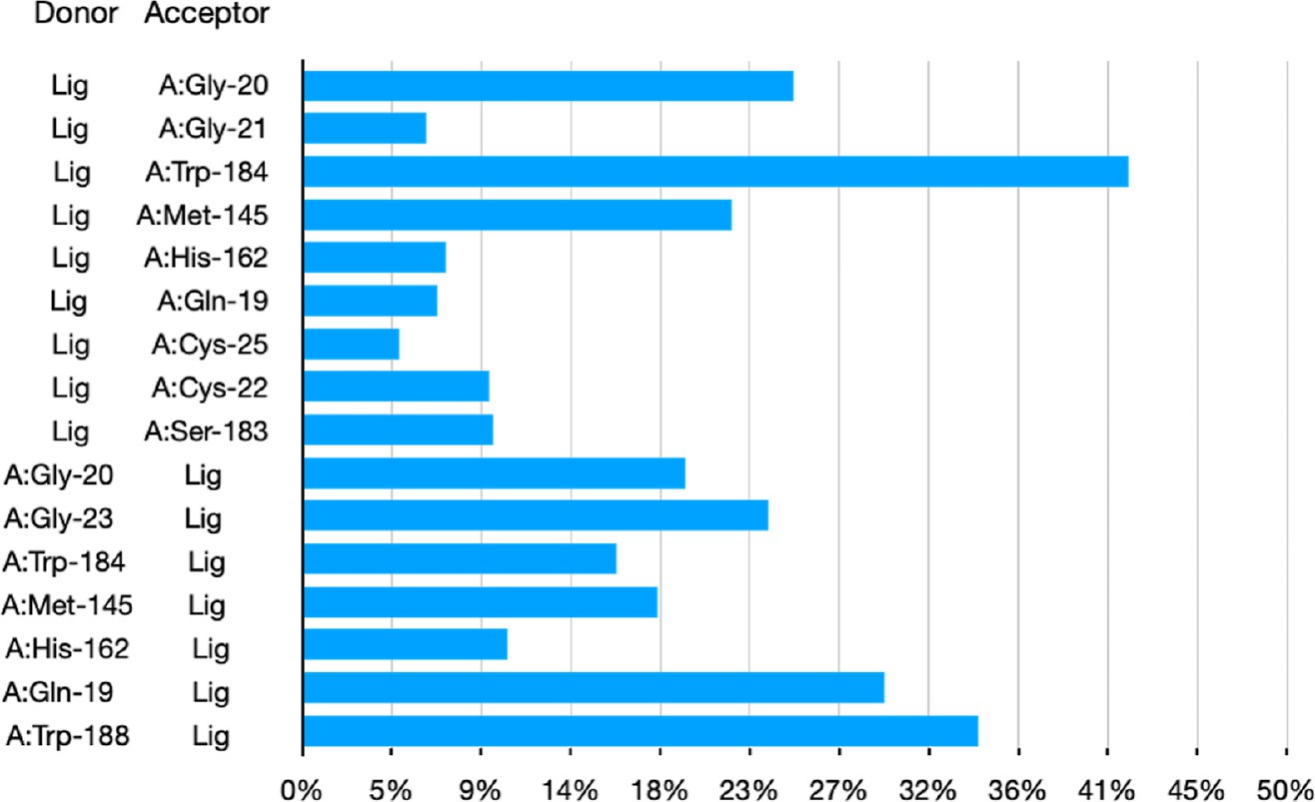
Frequency of hydrogen bonds (occupancy) between the CPN4F ligand and the cruzain receptor.

### 3.7 Drug Likeness Evaluation

In evaluating the degree of drug likeness of chalcone, it was observed that the CPN4F analog resides in a physicochemical space formed by compounds with moderate to high lipophilicity, with a calculated log *P* between 3.0 and 5.0 (Wlog *P* descriptor). This is within the lipophilicity threshold for good oral bioavailability according to Lipinski’s “rule of five”.^73^ Lipinski’s early experiments revealed that compounds within the threshold defined by log *P* ≤ 5, MW ≤ 500 g/mol, H-bond donors (HBD) ≤ 5, and H-bond acceptors (HBA) ≤ 10 had oral bioavailability in rats, as observed by permeability in Caco-2 cells.^73^

Pearson’s linear correlation with *r* = −0.881 indicates a strong inverse correlation between lipophilicity (Wlog *P*) and polar surface area (TPSA). A calculated TPSA of less than 140 Å^2^ ensures good distribution through cell membranes (Figure 27A).^106,107^ This suggests that the chalcone has an optimal alignment between solubility and permeability when compared to other drugs and enzyme inhibitors active against *T. cruzi* (BZN, nifurtimox, QUM, KB2, and BRZ). In this particular instance, QUM exhibited a log *P* >5 violation, thereby indicating a reduced propensity for development as an oral drug, in accordance with Lipinski’s rule.^73^

**Figure 27:**
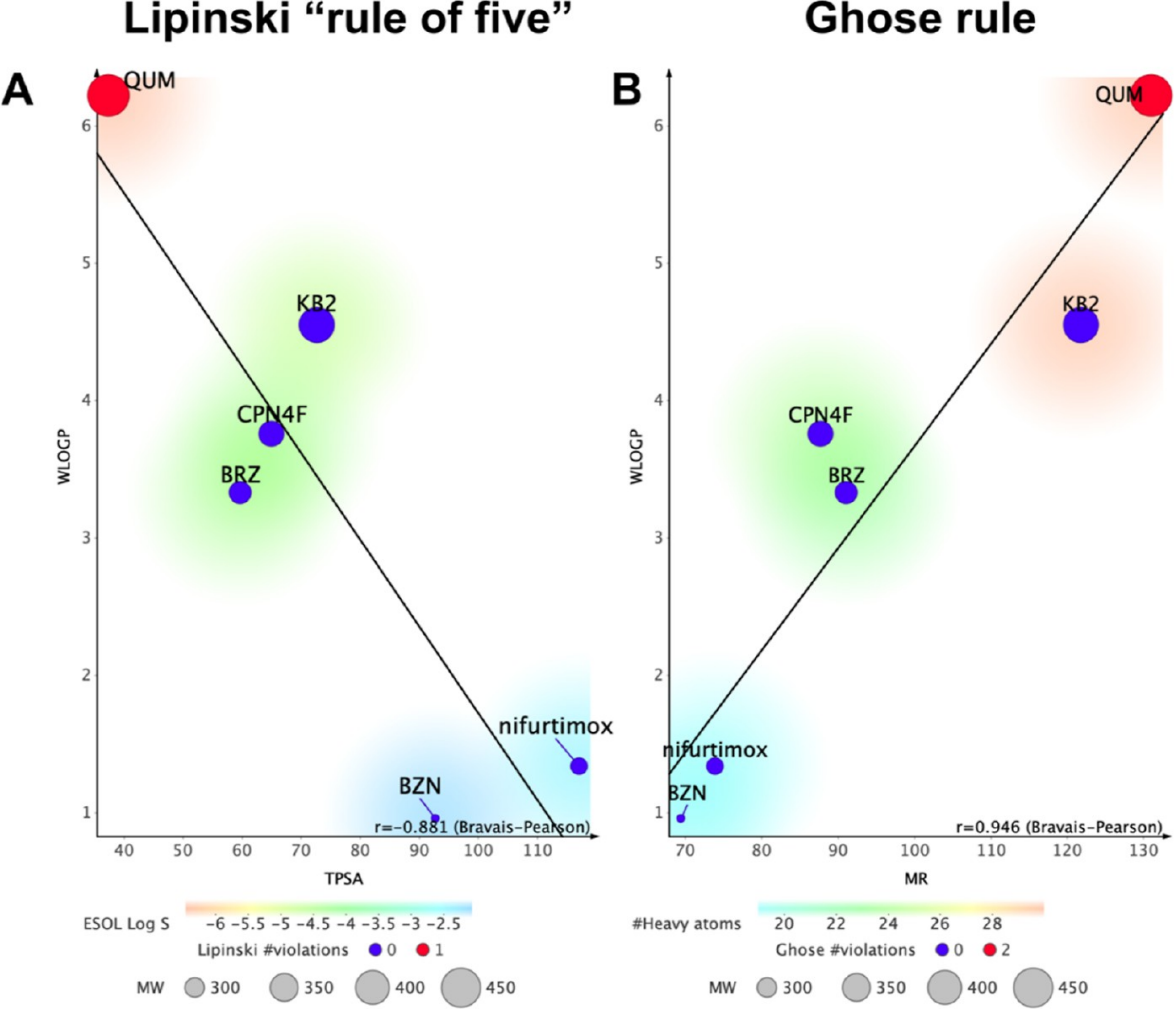
Predicted drug likeness properties for CPNAF in relation to known inhibitors of *T. cruzi* metabolic pathways, considering the criteria of (A) Lipinski’s “rule of five” and (B) Ghose rule.

Ghose’s rule, which considers the influence of molar refractivity (MR) on the polarizability of the molecule, showed a greater distinction between the compounds. In this criterion, CPN4F is in the physicochemical space of lead likeness together with BRZ (TcGAPDH inhibitor), indicating a pharmacophore similarity, preferably associated with the Michael receptor existing in both compounds, which consists of a carbonyl conjugated to an α,β-unsaturated system.^108^ The alignment between lipophilicity (Wlog *P*) and MR resulted in a Pearson linear correlation index of *r* = 0.946, indicating a direct proportionality between the two quantities. An increase in lipophilicity implies an increase in MR and polarizability (Figure 27B). Chalcone exhibited an MR of 87.71 (see Figure 27B), which demonstrates the order of hydrophobicity and van der Waals dispersive interactions, as well as a total of 24 heavy atoms (12 aromatic), within a MW range between 160 and 480 g/mol (see Figure 27B). The MR range (40–130) indicates that the compound exhibits an optimal alignment between molecular volume and polarizability, thereby promoting permeability, solubility, and oral absorption.^74^

In all drug likeness benchmark tests, the compound QUM (tryptophan reductase inhibitor) showed a higher number of violations. The implications in docking suggest low target binding specificity. This physicochemical distance from CPNF4F may be directly related to the low susceptibility of the protein to inhibition by chalcone. On the other hand, chalcone showed a drug likeness spectrum in relation to compounds KB2 and BRZ, inhibitors of calcineurin and trypanothione reductase, respectively, which may be related to the more favorable interactions that the compound showed against the enzymes.

### 3.8 MPO-Based ADME and Toxicity Prediction

For this study, MPO was applied to analyze a series of physicochemical properties systematically pertinent to the pharmacokinetic attributes commonly tangible in in vitro tests. According to Pfizer, Inc.,^34,109^ fluoro-substituted weak acids that are poorly lipophilic (log *P* < 3) that are larger and more polar than commonly marketed CNS-acting substances (TPSA > 20–40 Å^2^) are more favored molecules for the development of new drugs, as they present a better alignment between safety (low toxicity) and in vitro ADME descriptors: high permeability (*P*_app,A→B_ > 10 × 10^–6^ cm/s), low efflux rate by P-gp, and low hepatic clearance (CL_int,u_ < 8.0 mL/min/kg).^34^ Experimental data reveal that this optimal range, for small molecules (MW < 360 g/mol), is associated with compounds with a balance between oral absorption and metabolic stability.^110^

The MLP map in Figure 28A shows that the *p*-fluorine substitution on ring B creates a region of low lipophilicity. However, its inductive electron-withdrawing effect results in a region of lower electron density between ring A and the α,β-unsaturated carbonyl system, increasing the lipophilic potential of the chalcone molecular surface. Thus, the calculated log *P* value of around 3.91 is indicative of the high lipophilicity of the compound, with a strong contribution from the *p*-fluorine group and the O–H···O=C intramolecular hydrogen bond (structural increment = 0.65). It can also be seen that the OH group attached to ring A at 8.76 gives CPN4F a weak acid characteristic, indicating a point of equivalence between the acid species and the conjugated base at pH levels around 8.7, which is shifted toward the formation of the O^–^ conjugated base (charge = −1.0) as the pH increases from this point (Figure 28B). This feature suggests that at physiological pH (pH of around 7.4), the distribution of the acid species is greater than that of the conjugated base, which does not tend to cause abrupt changes in the lipophilic state of the compound.

**Figure 28:**
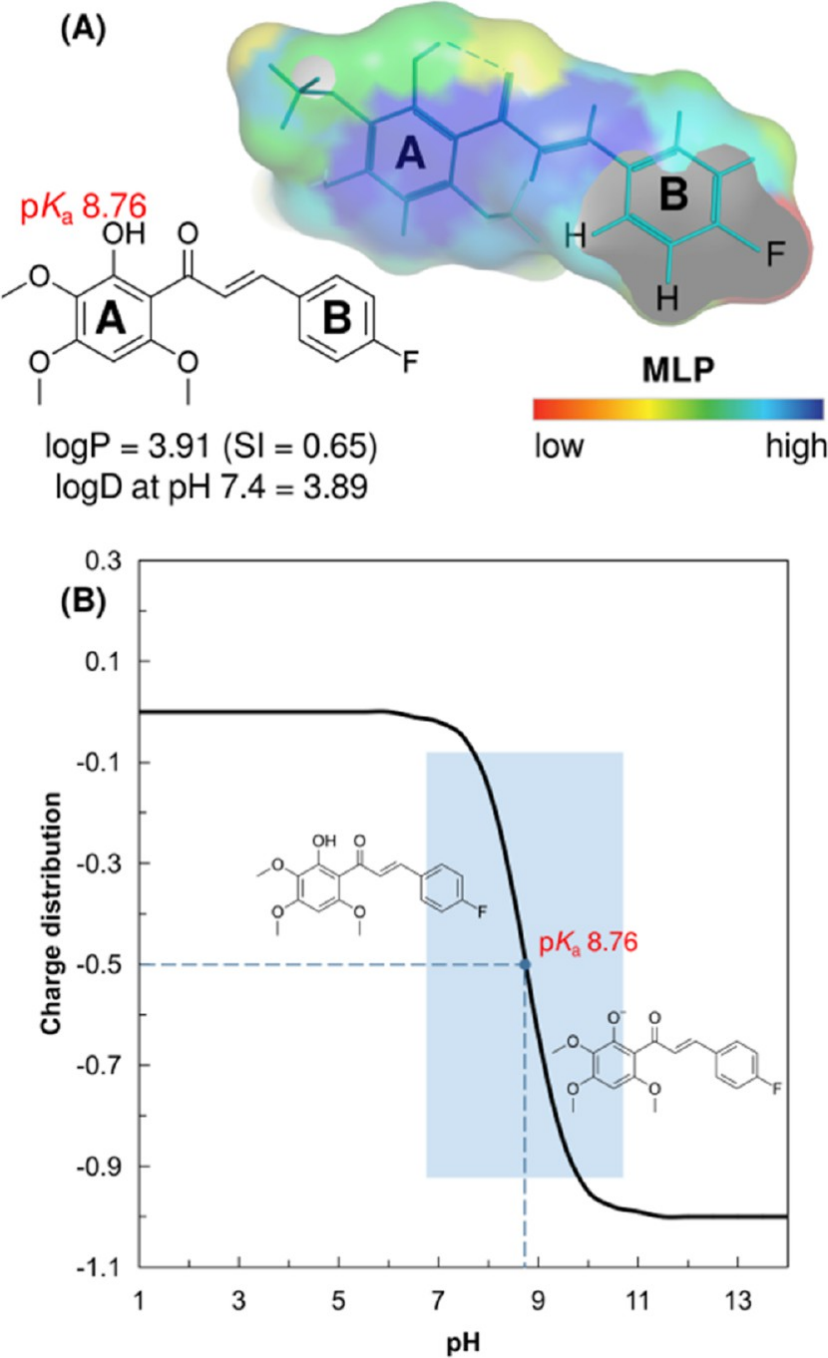
(A) Molecular lipophilicity potential (MLP) map and (B) conjugated acid–base equilibrium curve of chalcone CPN4F.

The MPO analyses indicate that high lipophilicity is the primary limiting factor in chalcone metabolism pharmacokinetics (Figure 29A). This is due to it being shifted outside the ideal spectrum expressed by the radar plot in Figure 29B (log *P*/log *D*), resulting in an MPO score of approximately 4. The value of 35 (on a scale of 0 to 6) falls within the ideal limit for an alignment between ADME attributes, which include passive permeability and hepatic clearance.^110^ When considering the calculated low topological polarity of 64.99 Å^2^, which is a product of the polar surfaces of the oxygenated methoxyl (–OCH_3_ = 9.23 Å^2^), carbonyl (–C=O = 17.07 Å^2^) and hydroxyl (–OH = 20.23 Å^2^) groups,^111^ it can be inferred that CPN4F is highly lipophilic and likely to act on the CNS (Figure 30A). However, the estimated range of lipophilicity in physiological environments (log *D* at pH 7.4 > 3.0) may limit the metabolic stability of the chalcone, within a molecular weight range of less than 360 g/mol (as shown in Figure 30B). This is consistent with the LipMetE descriptor, which indicates a low metabolic efficiency dependent on lipophilicity (as shown in Table 10).

**Figure 29:**
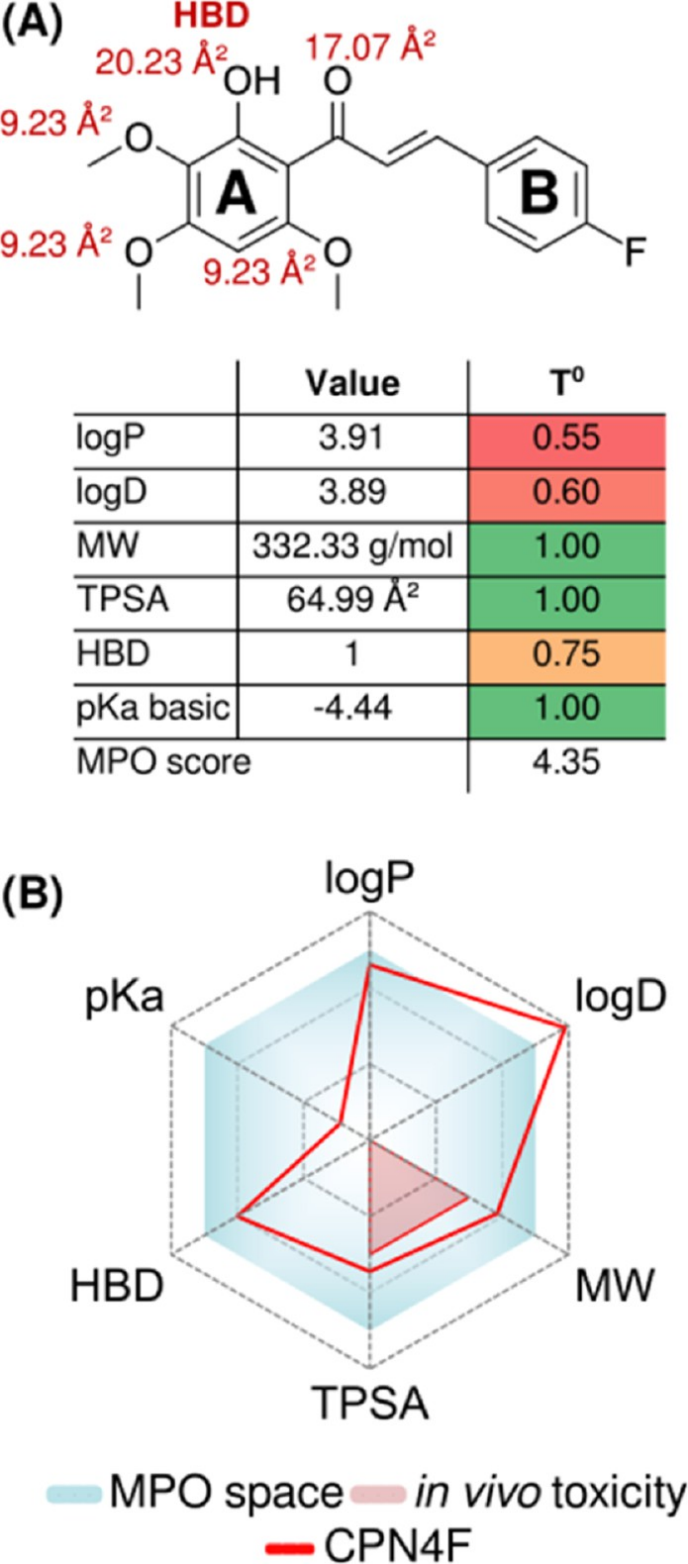
(A) Topological analysis and the transformed values (*T* ^0^) within the MPO algorithm’s desirability functions and (B) adjustment of the calculated properties to the MPO spectrum.

**Figure 30:**
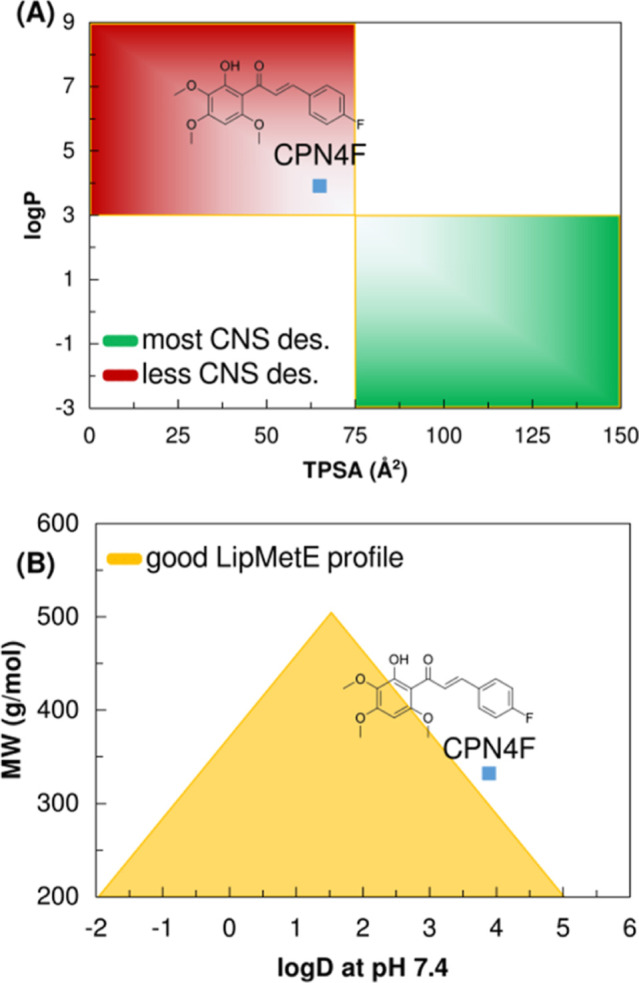
MPO analysis to estimate the (A) distribution of CPN4F to the CNS (log *P* vs TPSA) and the (B) alignment between permeability and metabolic stability (MW vs log *D* at pH 7.4).

**Table 10:** Predicted Binding Free Energy (kcal/mol) and Individual Energy Terms of the CP4NF-CRUZAIN Complex

term	CP4NF-CRUZAIN (kcal/mol)
*E*_vdW_	–24.85
*E*_ele_	–1.69
*G*_GB_	13.75
*G*_SA_	–3.05
–*T*Δ*S*	4.98
Δ*G*_bind_	–10.86 ± 3.89

The MPO analyses support the predicted ADME descriptors, as the estimated value of *P*_app,A→B_ = 1.5 × 10^–5^ cm/s indicates high passive permeability. However, the CL_int,u_ value of around 9.15 mL/min/kg indicates moderate clearance, reflecting the low metabolic stability of CPN4F. On the other hand, the compound’s low susceptibility to being a P-gp substrate suggests that it has greater permeability than passive efflux. This results in a predicted oral bioavailability (F %) of at least 94% (Table 11).^112–114^

**Table 11:** Pharmacokinetic Descriptors Predicted by the In Silico ADME Test and Toxicity Prediction Data

ADME property	predicted value	source
LipMetE	3.73	^72^
*P*_app,A→B_	1.5 × 10^–5^ cm/s	ADMETlab 2.0
log *P*_app_ em 10^–6^ cm/s	1.1	pkCSM
P-gp (substrate)	no	pkCSM
*F* %	94.53%	pkCSM
*F*_<30%_	0.005	ADMETlab 2.0
Fu	0.10	pkCSM
log BB	0.34	pkCSM
CYP1A2 (substrate)	0.94	ADMETlab 2.0
CYP2C9 (substrate)	0.93	ADMETlab 2.0
CYP2D6 (substrate)	0.88	ADMETlab 2.0
CYP3A4 (substrate)	0.47	ADMETlab 2.0
CL_int,u_	9.15 mL/min/kg	ADMETlab 2.0
log CL em mL/min/kg	0.16	pkCSM
DILI (probability)	0.57	ADMETlab 2.0
DILI (categorical)	no	pkCSM
mutagenic (probability)	0.07	ADMETlab 2.0
mutagenic (categorical)	no	pkCSM
LD_50_ (rat)	2100 mg/kg	ProTox-II
Regra da SureChEMBL	1 alerta (poly methoxyphenyl)	^73^

Furthermore, the substance’s high lipophilicity is strongly correlated with its estimated systemic distribution. This may limit its free unbound fraction in the blood plasma (Fu ∼ 0.1) and result in widespread distribution throughout biological tissues, including the blood–brain barrier (BBB). The log BB coefficient of 0.34 suggests that chalcone may be distributed to the central nervous system (CNS)^115^ (Table 11).

Figure 31A displays the 2D sensitivity map, which illustrates the correlation between the LipMetE descriptor and the prediction of metabolism sites and the identification of toxicophores. It is important to note that the presence of –OCH_3_ groups led to three CYP450-dependent *O*-dealkylation sites in phase I metabolism.^116^ These sites were mainly affected by the CYP1A2, CYP2C9, and CYP2D6 isoforms. The fragment was also identified by the SureChEMBL similarity test, which showed that CPN4F had strong similarity to a variety of patent-registered polymethoxychalcones (refer to Table 10). Furthermore, the compound possesses a preferred –OH group that undergoes glucuronidation by UDP-glucuronosyltransferase (UGT) during phase II metabolism,^117,118^ resulting in a significantly more polar and water-soluble metabolite that can be excreted. The compound was found to have a structural similarity of at least 79% with that of other compounds in the database (Figure 31B).

**Figure 31:**
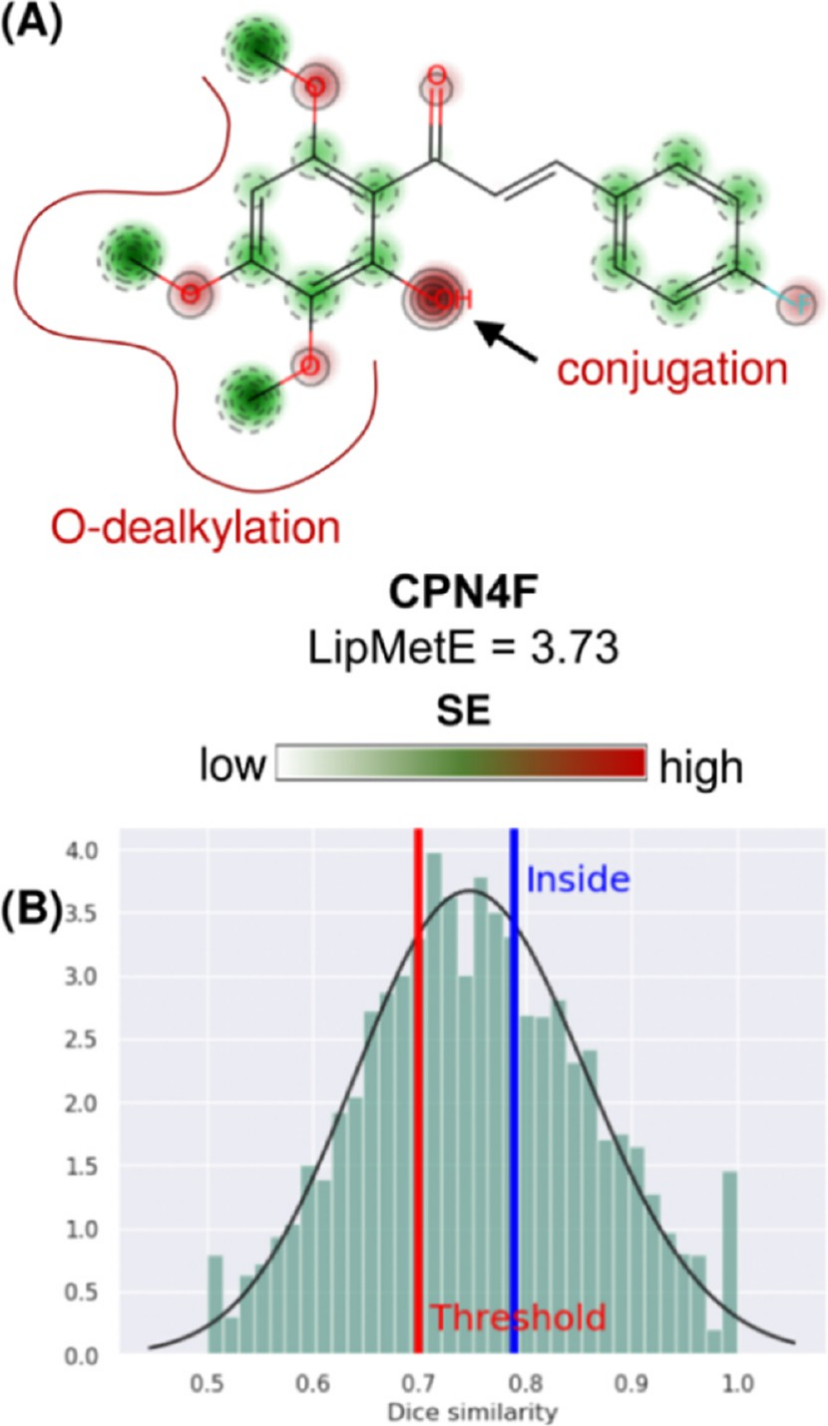
(A) Sensitivity map (SE) for metabolism site prediction and (B) 2D similarity of CPN4F with compounds from the database.

In predicting toxicity descriptors, it was observed that despite the low metabolic stability of CPN4F, the compound does not tend to form reactive metabolites in the human liver. This is mainly supported by the predictive models of drug-induced liver damage (DILI) and mutagenicity. The LD_50_ estimate of around 2100 mg/kg indicates that the compound belongs to toxicity class 5, where lethality of at least 50% of a tested species is unlikely^119^ (Table 11).

## 4 Conclusions

The experiments demonstrate that CPN4F can significantly reduce the survival of *T. cruzi*-infected cells. Increasing the amount of CPN4F tested resulted in a strong inhibition of parasite growth within the host cells. The electronic properties were characterized, and the chalcone CPN4F behaves primarily as an electrophile due to its strong acceptor tendency when analyzing the distribution of the LUMO, the electronic and condensed Fukui functions, the multiphilic index and the dual descriptor, and the reactivity descriptors, which include higher values for the electrophilic index, electronegativity, and global hardness.

The low SI values observed for CPN4F suggest a limited degree of selectivity with respect to the parasite. However, molecular docking simulations revealed that affinity energies ≤−6.0 kcal/mol were more significant in comparing the bioactivity of CPN4F in relation to BZN and the cruzain inhibitor KB2. This is due to the fact that the known inhibitors exhibited better selectivity of binding to the targets trypanothione reductase and TcGAPDH. Molecular dynamics simulations showed that the CPN4F–cruzain complex maintains its structure over 500 ns, with low rmsd changes and variations limited to the ends. MM/GBSA calculations revealed a binding energy of −10.86 kcal/mol, dominated by van der Waals interactions, and hydrogen bond analysis highlighted Trp-184 as a key residue for stabilization. These findings support the docking results and show that CPN4F strongly binds to cruzain. This suggests that it could be used to develop new cruzain inhibitors and therapies for Chagas disease.

The main limitation of this study is that it used a molecular docking model based on rigid proteins. This model is associated with a low degree of fidelity in the representation of the intracellular solvation system of the infected host cells. In this system, simulated proteins (cruzain, trypanothione reductase, and TcGAPDH) are expressed. On the other hand, ADME analyses showed that the compound can cross cell membranes and does not strongly bind to water, which suggests that it can interact with the active site of calcineurin, a target that has shown the most promising antiproliferative pharmacodynamic profile.

Although the present study demonstrates encouraging results regarding the potential of chalcone-based therapy for Chagas disease, it is important to note that further research is necessary to substantiate its medicinal efficacy. This includes conducting in vivo pharmacokinetic tests, such as the parallel artificial membrane permeability assay (PAMPA), and assessing metabolic stability in the human liver microsome (HLM) system. However, it should be noted that the scope of this study does not extend to these additional analyses.

## Supplementary Material



## Notes

Table S1: types of interaction between the CPN4F and BZN ligands in relation to the inhibitors cocrystallized in their respective targets: QUM-T.reductase, KB2-cruzain, and BZR-TcGAPDH (PDF)
