# Supplementary material for: Evaluation of Chalcone (E)‑3-(4-Fluorophenyl)-1-(2-hydroxy-3,4,6-trimethoxyphenyl)-prop-2-en-1-one against Trypanosoma cruzi (Y Strain): In Vitro Assay, In Silico Drug-Target Analysis, and MPO-Based Drug Likeness
Source: ACS Omega. 2026 Jul 23;11(30):44583–613. doi: 10.1021/acsomega.5c08163 (PMC13448934; doi:10.1021/acsomega.5c08163)
Supplement: Supplementary file 1 [file ao5c08163_si_001.pdf]

Evaluation of Chalcone (E)-3-(4-fluorophenyl)-1-(2-hydroxy-3,4,6-trimethoxyphenyl)-prop-2-en-1-one against *Trypanosoma cruzi* (Y strain): in vitro assay, in silico drug-target analysis and MPO-based drug likeness

[a]José Ivo Lima Pinto Filho, [b]Francisco Nithael Melo Lucio, [c]Lyanna Rodrigues Ribeiro, [d]Matheus Nunes da Rocha, [d,e]Hélcio Silva dos Santos, [f]Francisco Wagner de Queiroz Almeida-Neto, [c]Emanuel Paula Magalhaes, [c]Alice Maria Costa Martins, [c]Ramon Róseo Paula Pessoa Bezerra de Menezes, [d,c]Márcia Machado Marinho, [a]Pedro De Lima-Neto, [a,d,g]Emmanuel Silva Marinho*

[a]Department of Analytical Chemistry and Physical Chemistry, Federal University of Ceará, Fortaleza, CE, Brazil

[b]Doctoral Program in Biotechnology, Northeast Biotechnology Network, State University of Ceará, Fortaleza, Ceará, Brazil

[c]Department of Clinical and Toxicological Analysis, Federal University of Ceará, Fortaleza, CE, Brazil

[d]Postgraduate Program in Natural Sciences, Sciences and Technology Center, State University of Ceará, Fortaleza, CE, Brazil

[e]Center for Exact Sciences and Technology, State University of Vale do Acaraú, Sobral, CE, Brazil

[f]Center for Education, Science and Technology of the Inhamuns Region, State University of Ceará, Tauá, CE, Brazil

[g]Postgraduate Program in Veterinary Sciences, State University of Ceará, Fortaleza, Ceará, Brazil

Supplementary data

Table S1. Types of interaction between the CPN4F and BZN ligands in relation to the inhibitors co-crystallised in their respective targets: QUM-T.reductase, KB2-Cruzain and BZR-TcGAPDH.

**Target (PDB)**	**Ligand**	**Interactions**	
		**Interaction**	**Residue (Distance)**
Trypanothione reductase (PDB: 1GXF)	CPN4F	Hydrophobic	Val 65A (3.77 Å),Gln 69A (3.47 Å),Pro 71A (3.64 Å)
		H-bond	His 73A (1.92 Å)
	BZN*	Hydrophobic	Lys 61A (3.87 Å), Ile 200A (3.75 Å)
		H-bond	Arg 288A (3.27 Å)

		π-stacking	Phe 199A (5.40 Å)
	QUM**	Hydrophobic	Leu 18A (3.90 Å), Trp 22A (3.47 Å), Trp 2A (3.49 Å), Met 114A (3.91 Å), Asp 17A (3.30 Å)
		π-stacking	Trp 22A (3.73 Å), Trp 22A (4.09 Å)
		Salt bridges	Glu 133A (4.96 Å), Asp 117A (4.91 Å)
Cruzain (PDB: 3IUT)	CPN4F	Hydrophobic	Gln 21A (4.70 Å), Trp 184A (3.99 Å), Trp 188A (5.95 Å)
		H-bond	Gln 19A (3.41 Å), Trp 184A (3.15 Å)
		π-stacking	Trp 184A (3.71 Å)
	BZN*	Hydrophobic	Trp 7A (3.80 Å), Arg 10A (3.97 Å), Arg 10A (3.86 Å), Gln 21A (4.06 Å), Phe 39A (3.55 Å), Leu 40A (3.59 Å)
		H-bond	Gln 19A (4.22 Å), Phe 39A (2.57 Å), Gly 42A (3.31 Å), Gly 42A (3.44 Å), Trp 84A (2.83 Å), Trp 188A (3.49 Å)
	KB2**	Hydrophobic	Leu 67A (3.88 Å), Leu 67A (3.59 Å)
		H-bond	Gln 19A (2.28 Å), Gly 23A (2.89 Å), Cys 25A (2.18 Å), Gly 66A (1.87 Å), Gly 66A (2.27 Å)
TcGAPDH (PDB: 1K3T)	CPN4F	Hydrophobic	Thr 197C (3.66 Å), Glu 336C (3.67 Å)
		H-bond	Cys 166C (2.49 Å), Thr 167C (2.94 Å), Thr 167C (2.44 Å), His 194C (2.46 Å), Thr 226C (2.11 Å)
	BZN*	Hydrophobic	Ile 731C (3.72 Å), Glu 1054C (3.64 Å)
		H-bond	His 912C (3.48 Å), Ser 965C (3.17 Å)
		π-stacking	His 912C (4.13 Å)
	BZR**	Hydrophobic	Thr 199C (3.90 Å), Asn 335C (3.47 Å)
		H-bond	Cys 166C (2.74 Å), Thr 167C (2.80 Å), Thr 167C (3.05 Å)
		Salt bridge	His 194C (3.95 Å)

*Reference drug, ** Inhibitor co-crystallised to the target in PDB.
